# Detection of Genus and Three Important Species of *Cronobacter* Using Novel Genus- and Species-Specific Genes Identified by Large-Scale Comparative Genomic Analysis

**DOI:** 10.3389/fmicb.2022.885543

**Published:** 2022-06-02

**Authors:** Lu Wang, Pan Wu, Yingying Su, Yi Wei, Xi Guo, Lan Yang, Min Wang, Bin Liu

**Affiliations:** ^1^Hubei Key Laboratory of Tumor Microenvironment and Immunotherapy, China Three Gorges University, Yichang, China; ^2^Institute of Infection and Inflammation, China Three Gorges University, Yichang, China; ^3^Medical College, China Three Gorges University, Yichang, China; ^4^TEDA Institute of Biological Sciences and Biotechnology, Nankai University, Tianjin, China; ^5^Key Laboratory of Molecular Microbiology and Technology, Ministry of Education, Nankai University, Tianjin, China; ^6^Key Laboratory of Dairy Biotechnology and Engineering, Ministry of Education, Key Laboratory of Dairy Products Processing, Ministry of Agriculture, Inner Mongolia Agricultural University, Huhhot, China

**Keywords:** *Cronobacter* spp., foodborne pathogen, genomic analysis, marker gene, PCR

## Abstract

The genus *Cronobacter* includes seven species; however, the strains of *Cronobacter sakazakii*, *Cronobacter malonaticus*, and *Cronobacter turicensis* were highly correlated with clinical infections. Rapid and reliable identification of these three species of *Cronobacter* is important in monitoring and controlling diseases caused by these bacteria. Here, we identified four pairs of novel marker genes for the *Cronobacter* genus, *C. sakazakii*, *C. malonaticus*, and *C. turicensis* based on large-scale comparative genomic analysis from 799 *Cronobacter* and 136,146 non-*Cronobacter* genomes, including 10 *Franconibacter* and eight *Siccibacter*, which are close relatives of *Cronobacter*. Duplex and multiplex PCR methods were established based on these newly identified marker genes. The reliability of duplex and multiplex PCR methods was validated with 74 *Cronobacter* and 90 non-*Cronobacter* strains. Strains of *C. sakazakii*, *C. malonaticus*, and *C. turicensis* could be detected accurately at both the genus and species level. Moreover, the newly developed methods enable us to detect 2.5 × 10^3^ CFU/ml in pure culture. These data indicate that the accurate and sensitive established methods for *Cronobacter* can serve as valuable tools for the identification of these strains recovered from food, environmental, and clinical samples.

## Introduction

*Cronobacter*, belonging to the family Enterobacteriaceae, is a genus of Gram-negative, motile, facultative anaerobic, opportunistic, foodborne pathogens that can cause bacteremia, meningitis, and necrotizing enterocolitis in neonates (Forsythe, 2018). *Cronobacter* has been isolated from various environments (Killer et al., 2015; Singh et al., 2015; Ling et al., 2018; Li et al., 2020), and several disease cases have been associated with the ingestion of *Cronobacter*-contaminated dry food products, such as powdered milk formula (Drudy et al., 2006; Forsythe, 2018). Despite the low incidence of infection, the mortality of *Cronobacter* infection in neonates can be as high as 27%–80% (Drudy et al., 2006; Masood et al., 2015). The *Cronobacter* species of serious clinical significance are *Cronobacter sakazakii*, *Cronobacter* malonaticus, and *Cronobacter turicencis*, and other four species of the genus (*Cronobacter universalis*, *Cronobacter dublinensis*, *Cronobacter muytjensii*, and *Cronobacter condimenti*) are primarily environmental commensals with low clinical significance (Sonbol et al., 2013; Feeney et al., 2014; Forsythe, 2018; Li et al., 2020). Thus, reliable methods to identify *C. sakazakii*, *C. malonaticus*, and *C. turicensis* are critical to reduce mortality and transmission of diseases caused by *Cronobacter* spp.

Molecular detection methods are more useful tools than traditional methods to increase our understanding of the epidemiology of a bacterium important to public health. These protocols are usually designed to amplify DNA fragments of certain genes contained in genomes of the pathogen of interest. Over the past decade, a range of molecular methods based on genes, such as *16S rDNA* (Malorny and Wagner, 2005), *23S rDNA* (Derzelle et al., 2007), *MMS* (Seo and Brackett, 2005), *rpoB* (Stoop et al., 2009), *ompA* (Zimmermann et al., 2014), *fusA* (Li et al., 2017), *cgcA* (Carter et al., 2013), *ygrB* (Huang et al., 2013), etc. have been developed to identify *Cronobacter* spp. These approaches can be used as alternatives to traditional culture-based detection methods or can be used to confirm results generated by traditional approaches. However, only a few of these methods are able to simultaneously detect and differentiate species within the *Cronobacter* genus. *rpoB* had been used to detect six species of *Cronobacter* (Stoop et al., 2009); however, a two-step PCR procedure was needed to differentiate between *C. sakazakii* and *C. malonaticus*. Even though *gyrB* (Huang et al., 2013) was utilized for direct species identification of *C. sakazakii* and *C. dubliniensis*, the method cannot distinguish between other species. Primers were designed based on *cgcA* (Carter et al., 2013) to identify *Cronobacter* at the species level; however, non-specific amplicons occurred. Thus, it is necessary to identify novel specific markers for *Cronobacter* spp. and develop efficient identification methods according to these markers.

As high-throughput genome sequencing technologies continue to improve, the number of sequenced microbial genomes has continued to increase dramatically over the past decade. This makes it possible to employ an *in silico* large scale comparative genomic approach coupled with *in vitro* PCR validation to facilitate the translation of genomic data into diagnostic marker gene discoveries. In this study, a low-cost and simple attempt was made to identify novel diagnostic marker genes specific for *Cronobacter* spp.

## Materials and Methods

### Bacterial Strains and Genome Sequences

A total of 164 bacteria isolates, including 74 *Cronobacter* strains (62 *C. sakazakii*, five *C. dubliniensis*, three *C. malonaticus*, two *C. turicensis*, and two *C. universalis*) and 90 non-*Cronobacter* strains (18 *Enterobacter cloacae*, 36 *Enterobacter aerogenes*, and 36 *Escherichia coli*), were used in this study for *in vitro* validation (Supplementary Table S1). Moreover, 799 *Cronobacter* (578 *C. sakazakii*, 100 *C. malonaticus*, 60 *C. dubliniensis*, 35 *C. turicensis*, 15 *C. muytjensii*, nine *C. universalis*, and two *C. condimenti*) and 136,146 non-*Cronobacter* genomes belonging to 31 genera were used for large-scale *in silico* comparative genomic analysis (Supplementary Tables S2, S3). These non-*Cronobacter* genomes include 10 *Franconibacter*, eight *Siccibacter*, and 810 *E. cloacae*, which are close relatives of genus *Cronobacter*.

### Phylogenetic Analysis

Single-copy core genes found using OrthoFinder v2.3.3 (Emms and Kelly, 2015) were used as original data for construction of a phylogenetic tree. *Enterobacter cloacae* ATCC 13047™ and ECNIH2 (GenBank accession number GCA_000025565.1 and GCA_000724505.1, respectively) served outgroups, as it is the species closely related to the *Cronobacter* genus (Joseph et al., 2012). MAFFT v7 with “G-INS-I” alignment method (Katoh and Standley, 2013) was used for creating multiple sequence alignments for each core gene and resulting alignments were concatenated. Thereafter, RAxML v8 with GTR (General Time Reversible) evolution model (Stamatakis, 2014) was applied to construct a phylogenetic maximum likelihood tree. The tree and subtrees were plotted with the R package’s ggtree (Yu et al., 2016). To confirm the degree of genomic relatedness and clarify relationships between the species of *Cronobacter*, ANIb values (the average nucleotide identity values based on BLAST) for all possible pairs of genomes were calculated using the program FastANI v.1.0 (Jain et al., 2018).

### Identification of Genus-Specific Genes for *Cronobacter*

Single-copy core genes in the *Cronobacter* genus were identified using OrthoFinder. Large-scale blast score ratio (LS-BSR) software was used to identify highly-conserved genes in the *Cronobacter* genus compared with other non-*Cronobacter* bacteria (Sahl et al., 2014). This was run against the assembled genomes of 799 *Cronobacter* isolates and 136,146 non-*Cronobacter* isolates belonging to 31 genera. Thereafter, the matrix generated by LS-BSR was processed using a script developed in house to evaluate and visualize the highly conserved genes across the data set. Genes with an average blast score ratio (BSR) value >0.9 in all *Cronobacter* genomes and <0.1 in all non-*Cronobacter* genomes were considered highly conserved genes in *Cronobacter*. These genes were further screened manually (genes with the smallest BSR value <0.8 in any *Cronobacter* genome or the largest BSR value >0.4 in any non-*Cronobacter* genome were excluded) and searched against the full National Center for Biotechnology Information (NCBI) nucleotide database to confirm their specificity. Two promising conserved genes were selected for identification of the *Cronobacter* genus.

### Identification of Species-Specific Genes of *Cronobacter* spp.

As shown above, highly conserved single-copy core genes in each species of *C. sakazakii*, *C. malonaticus*, and *C. turicensis* compared with other non-*Cronobacter* species were identified using OrthoFinder and LS-BSR. To explore whether these highly conserved genes were specific for target species and absent in the other six species of *Cronobacter*, these genes were analyzed using LS-BSR once more. Moreover, these genes, with an average BSR value > 0.9 in target species genomes and <0.4 in the other six *Cronobacter* species genomes, were considered as candidate marker genes. These candidate marker genes were screened manually once more (genes with the smallest BSR value < 0.8 in all *Cronobacter* genomes or largest BSR value > 0.4 in all non-*Cronobacter* genomes were excluded) and searched against the NCBI nucleotide database to confirm their specificity. The two most promising conserved genes of each species were selected for identification of *C. sakazakii*, *C. malonaticus*, and *C. turicensis*.

### PCR Primers

Multiple sequence alignment of genus- and species-specific gene alleles was performed to obtain conserved regions, which were used for primer design by Primer Premier 6.0 (Supplementary Figure S1). Thereafter, the specificity of theory sequences of amplicons was verified using BLAST and primer sequences were evaluated for their ability to form homo- and heterodimers as well as hairpins using the oligo-analyzer. Desalted primers were synthesized from Invitrogen. Primer sequences and corresponding theory amplicon sizes are shown in Table 1.

**Table 1:** Primer sets utilized in PCR assay and their target genes.

Primer set	Reference Genome	Marker gene	Description	Location	Primer name	Concentration	Primer sequence (5′–3′)	Amplicon size
Cro_set (for *Cronobacter*)	ATCC 29544™	*yifL*	Predicted small periplasmic lipoprotein	742,985–742,782	croP1F^#^	150 nM	TTACTTCCCGCCAGCAGAC	94 bp
					croP1R^#^	150 nM	ATCGCCACGGTTGTTGGT	
		*ygcB*	Hypothetical protein	1,477,530–1,477,252	croP2F	200 nM	GCTTACCGCCAGCATGGT	228 bp
					croP2R	200 nM	ACTTCCACCATGACGTCTTT	
Sak_set (for *C. sakazakii*)	ATCC 29544™	*fimG*	Type 1 fimbria component protein	3,972,871–3,973,380	sakP1F	125 nM	GACGATATCAACCTGCAG	239 bp
					sakP1R	125 nM	CTGTAAGCCGTACTGTTAGTCG	
		*nanK*	Predicted N-acetyl mannosamine kinase	600,571–599,696	sakP2F^#^	300 nM	GTACTGGCGATAGACATAGGTGG	525 bp
					sakP2R^#^	300 nM	GATAGCCTCCACACACCCTG	
Mal_set (for *C. malonaticus*)	LMG 23,826	*papD*	Pili assembly chaperone	2,969,163–2,968,486	malP1F^#^	200 nM	GCCTCATTATCGGTGCAGAAT	342 bp
					malP1R^#^	200 nM	CAGTTTATCCGTTGCCGATT	
		*sthD*	Fimbrial protein	2,965,864–2,965,289	malP2F	175 nM	TCAGAGCATGGCGGCAGGAA	108 bp
					malP2R	175 nM	GTTCCAAGCTTCACCACGCCTG	
Tur_set (for *C. turicensis*)	z3032	*phpB*	Adenosylcobalamin/alpha-ribazole phosphatase	1,339,511–1,338,891	turP1F	200 nM	CGCGTCTGAACGAGATGTT	294 bp
					turP1R	200 nM	GCTCCAGCAGGAAATGCC	
		*nudI*	Nucleoside triphosphatase	2,983,801–2,983,376	turP2F^#^	200 nM	TGTCCTGTGATACAAAATGATGG	155 bp
					turP2R^#^	200 nM	CCTAACTCCTCCATTATTTCACG	

# Primer set (CroM_set) used to establish multiplex PCR assay.

### Verification of Specificity by Duplex and Multiplex PCR Assays

Specificity of each primer set was assessed by running a PCR assay on a panel of bacterial strains consisting of 74 *Cronobacter* and 90 non-*Cronobacter* isolates. PCR mixtures contained 10 μl Ex Taq Master Mix (Takara, the premixed solution contains Ex Taq DNA Polymerase, 2 × PCR Buffer, and 200 μM dNTPs), 0.5 μl of each primer (optimal concentration shown in Table 1), 1 μl bacterial template (1 × 10^6^ CFU/ml), and RNase/DNase free water to adjust the volume to 20 μl. All PCR runs included a negative control without template. PCR reactions were run as follows: initial hot start (94°C for 10 min), amplification for 35 cycles (94°C for 30 s, 59.5°C for 30 s, and 72°C for 60 s), and final extension (72°C for 10 min). Annealing temperatures were optimized by gradually increasing the temperature from 50 to 65°C in the assay. PCR products were examined using agarose gel electrophoresis and visualized after ethidium bromide staining. For sensitivity verification, pure cultures of *C. sakazakii* ATCC 29544™, *C. malonaticus* LC03, and *C. turicensis* LC08 with a concentration of 2.5 × 10^6^ CFU/ml were serially diluted 10-fold to 2.5 × 10^2^ CFU/ml and used as PCR templates.

## Results

### Phylogenetic Analysis

**Figure 1:**
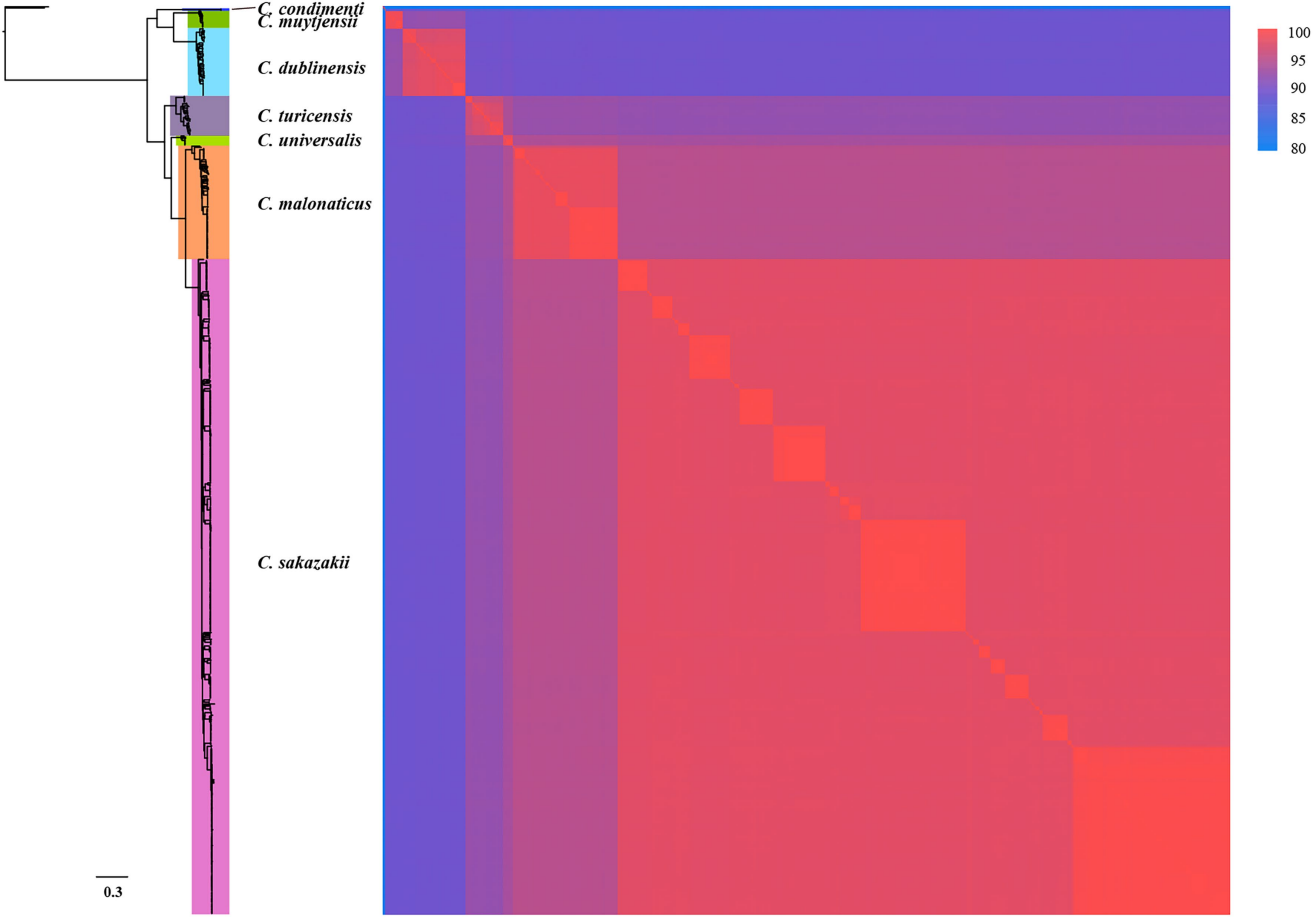
Whole-genome phylogeny of seven species of *Cronobacter*. The maximum-likelihood phylogeny tree was constructed based on single-nucleotide polymorphisms extracted from 799 genomes, excluding those resulting from recombination (two *Enterobacter cloacae* strains were used as the outgroup). ANIb values of the 799 *Cronobacter* genomes was calculated using FastANI.

To confirm that 799 genomes belonged to corresponding species of *Cronobacter*, a maximum-likelihood phylogenetic tree was constructed, based on 223 single-copy core genes. Phylogenetic analysis showed that the genus was divided into seven clades corresponding to seven species of *Cronobacter* (Figure 1). To explore the genomic similarities among phylogenetic clades further, ANIb values of all genome assemblies were calculated using fastANI (Figure 1). All intraclade ANIb values exceeded the commonly used 95% species-level threshold (Kalyantanda et al., 2015) for *C. sakazakii* (97.29%–99.99%), *C. malonaticus* (96.33%–99.99%), *C. turicensis* (95.99%–99.99%), *C. dubliniensis* (96.91%–99.99%), *C. muytjensii* (98.72%–99.99%), *C. universalis* (98.20%–99.99%), and *C. condimenti* (99.93%–99.97%), showing that each clade represented a single species.

### Identification of *Cronobacter* Genus-Specific Marker Genes

**Figure 2:**
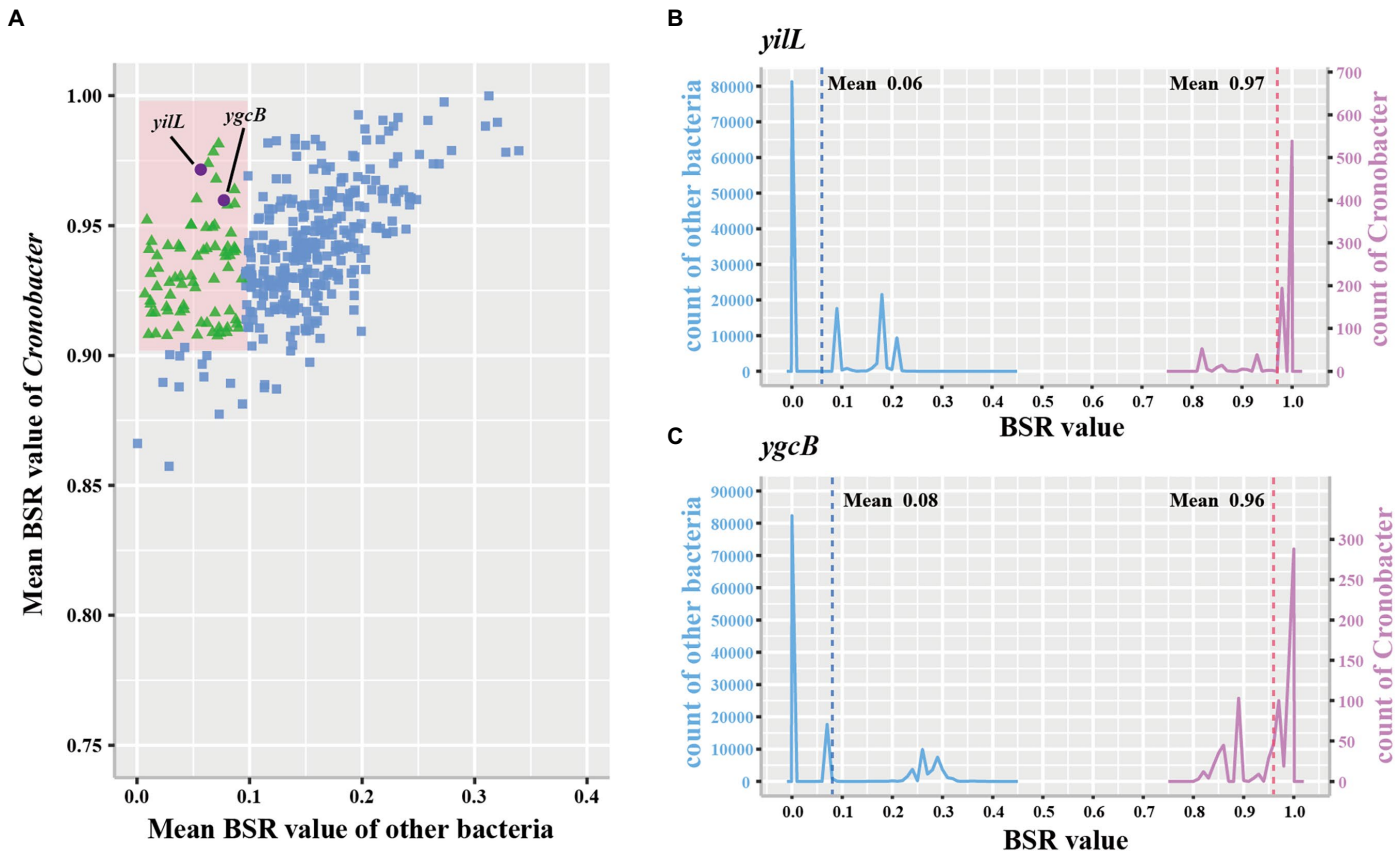
Conserved single-copy core genes of *Cronobacter* were further evaluated by large-scale blast score ratio (LS-BSR). **(A)** LS-BSR analysis of each conserved genes among *Cronobacter* and non-*Cronobacter* bacteria. **(B,C)** BSR value distribution of the *Cronobacter*-specific marker genes *yifL* and *ygcB*.

Three hundred and ninety-one conserved single-copy core genes were identified from 799 accessible *Cronobacter* genomes in the PubMLST database using OrthoFinder. To explore whether these conserved single-copy core genes were specific for the *Cronobacter* genus, we used the large-scale BLAST score ratio (LS-BSR) to evaluate genes present in the 799 isolates of the *Cronobacter* genus yet absent in 136,146 isolates, belonging to 31 genera of non-*Cronobacter* common environmental microbes and pathogens. According to the BSR value, 78 genes were highly conserved in *Cronobacter* species (average BSR value > 0.9 in *Cronobacter* and <0.1 in non-*Cronobacter*; Figure 2A). To find genes that uniquely existed in all *Cronobacter* isolates and deficient in all other non-*Cronobacter* bacteria, genes with a BSR value <0.8 in any *Cronobacter* isolates and >0.4 in any non-*Cronobacter* isolates were excluded. We finally selected two most promising conserved genes (*yifL* and *ygcB*) as *Cronobacter* genus-specific marker genes after manual screening and searching against the NCBI nonredundant nucleotide database (Figures 2B,C). Characteristics of the two genus-specific genes and corresponding designed primers are shown in Table 1.

### Identification of Species-Specific Marker Genes of *Cronobacter sakazakii*, *Cronobacter malonaticus*, and *Cronobacter turicensis*

**Figure 3:**
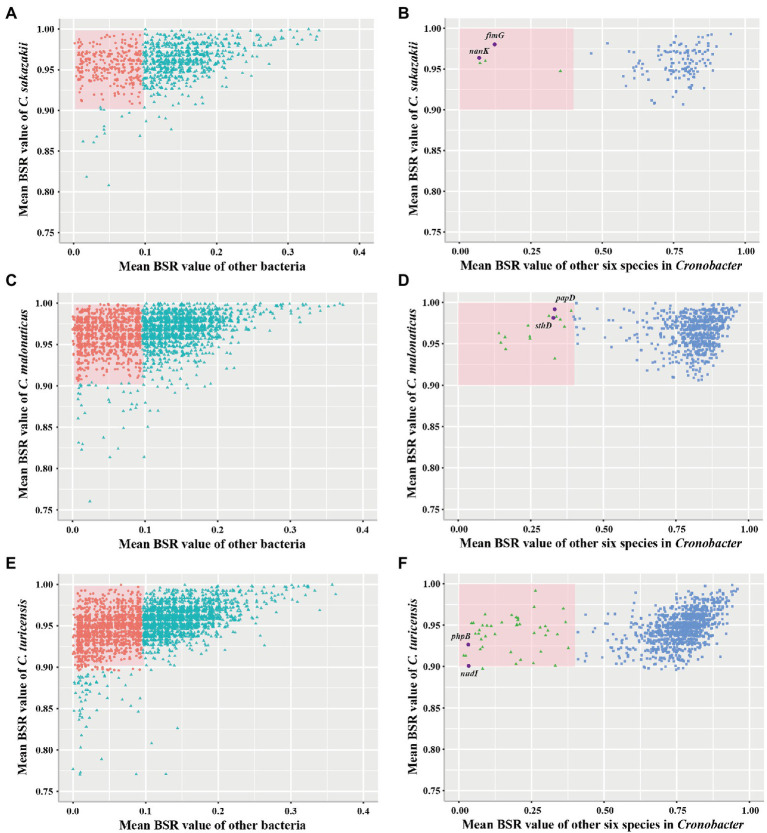
Conserved single-copy core genes of *Cronobacter sakazakii*, *Cronobacter malonaticus*, and *C. turicensis* were further evaluated by LS-BSR. **(A,C,E)** LS-BSR analysis of each conserved genes among target species and non-*Cronobacter* bacteria. **(B,D,F)** LS-BSR analysis of each gene analyzed again among target species and other six species of *Cronobacter*.

To examine whether marker genes exist in *C. sakazakii*, *C. malonaticus*, and *C. turicensis*, further analysis was performed. A total of 1,002, 2,555, and 3,238 conserved single-copy core genes were identified from 578 *C. sakazakii*, 100 *C. malonaticus*, and 35 *C. turicensis* genomes using OrthoFinder, respectively. Using LS-BSR, we discovered 134, 683, 1,110 genes that were conserved in *C. sakazakii*, *C. malonaticus*, and *C. turicensis* yet absent in other non-*Cronobacter* bacteria (Figures 3A,C,E; using a threshold of the average BSR value >0.9 in each target species and <0.1 in non-*Cronobacter* bacteria). To identify genes that were only conserved in genomes of each target species, the LS-BSR comparison of genomes between each target species and the remaining six species of *Cronobacter* demonstrated that 5, 14, 44 genes were highly conserved in *C. sakazakii*, *C. malonaticus*, and *C. turicensis*, respectively (Figures 3B,D,F). To confirm specificity, these candidate genes were screened manually and searched against the NCBI nonredundant nucleotide database and the two most promising conserved genes were selected as species-specific marker genes for each species (*fimG* and *nanK* for *C. sakazakii*, *papD* and *sthD* for *C. malonaticus*, and *phpB* and *nudI* for *C. turicensis*; Supplementary Figures S2–S4). Characteristics of the three pairs of species-specific genes and corresponding designed primers are shown in Table 1.

### Specificity Evaluation Using the Duplex PCR Assay

**Figure 4:**
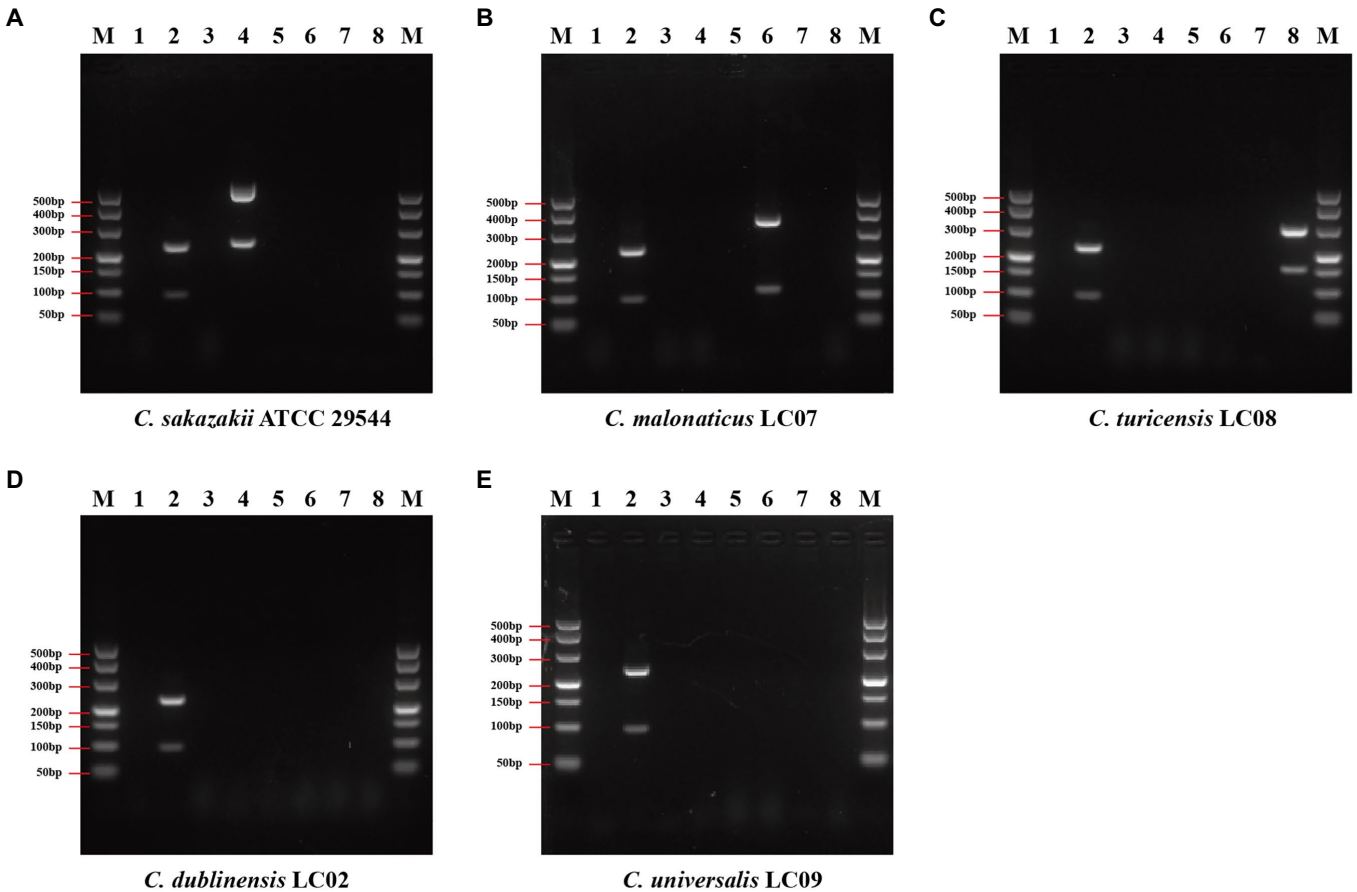
Results of duplex PCR based on genus- and species-specific genes. **(A)** *Cronobacter sakazakii* ATCC 29544™, **(B)** *Cronobacter malonaticus* LC07, **(C)** *C. turicensis* LC08, **(D)** *C. dubliniensis* LC02, and **(E)** *Cronobacter universalis* LC09. Lane M: DNA marker; lanes 1, 3, 5, and 7: negative control without template; lane 2: *Cronobacter* genus primer set Cro_set; lane 4: *C. sakazakii* species primer set Sak_set; lane 6: *C. malonaticus* species primer set Mal_set; and lane 8: *C. turicensis* species primer set Tur_set.

The duplex PCR assay was developed based on genus or species marker genes to evaluate the specificity of designed primers. Seventy-four *Cronobacter* strains and 90 non-*Cronobacter* strains, closely related to *Cronobacter*, were utilized to evaluate specificity of genus- and species-specific primer sets (each primer set contains two primer pairs, shown in Table 1). The specificity of each primer set was crosstested with isolates of target species of *Cronobacter* and non-target species of *Cronobacter* and non-*Cronobacter*. The results showed that each primer set successfully amplified their target genes with correct amplicon sizes and without non-specific band (Figure 4; Supplementary Table S4). Primer set Cro_set could detect all isolates of *Cronobacter*, and primer sets Sak_set, Mal_set, and Tur_set could only detect corresponding isolates from *C. sakazakii*, *C. malonaticus*, and *C. turicensis*, respectively (Figure 4; Supplementary Table S4). To confirm specificity, 90 non-*Cronobacter* strains were tested and all produced no PCR products (Supplementary Table S4).

### Specificity Evaluation Using the Multiplex PCR Assay

**Figure 5:**
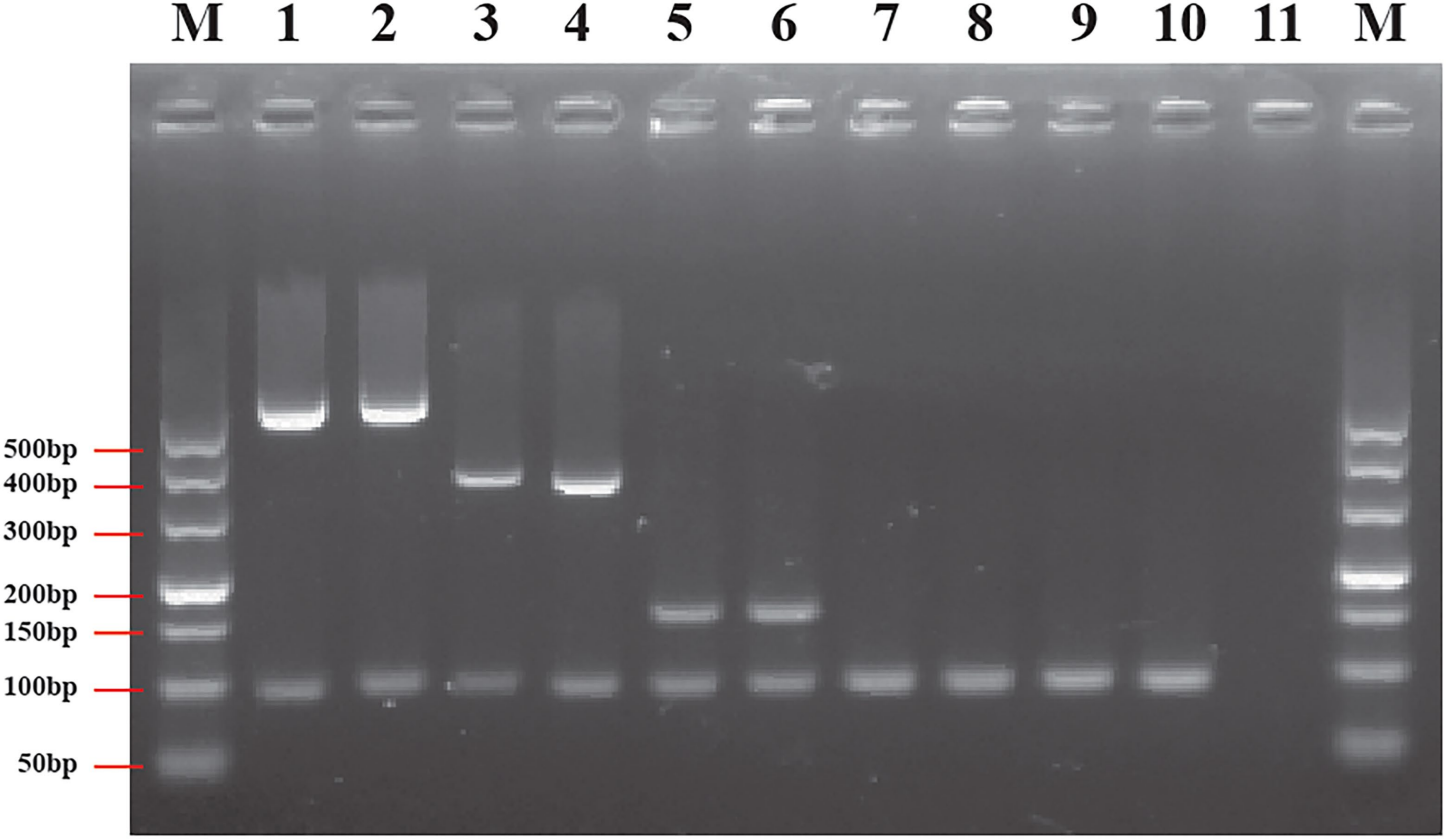
Results of multiplex PCR based on genus- and species-specific genes. Lane M: DNA marker; lane 1: *Cronobacter sakazakii* ATCC 29544™; lane 2: *C. sakazakii* ATCC BAA-894™; lane 3: *Cronobacter malonaticus* LC03; lane 4: *C. malonaticus* LC07; lane 5: *Cronobacter turicensis* LC08; lane 6: *C. turicensis* LC12; lane 7: *Cronobacter dubliniensis* LC01; lane 8: *C. dubliniensis* LC02; lane 9: *Cronobacter universalis* LC09; lane 10: *C. universalis* LC10; and lane 11: negative control without template.

To differentiate species of *Cronobacter* using only one PCR reaction accurately, a multiplex primer set CroM_set (shown in Table 1), including four specific primer pairs, croP1F/croP1R, sakP2F/sakP2R, malP1F/malP1R, and turP2F/turP2R, were selected based on amplicon sizes to develop the multiplex PCR assay. Primer pairs were mixed and used to screen 2.5 × 10^5^ CFU/ml pure culture from 74 *Cronobacter* isolates. The results showed that all target isolates produced the expected PCR products and non-target isolates gave no PCR products (Supplementary Table S4). Representative PCR results using 10 *Cronobacter* pure cultures as templates are shown in Figure 5. To confirm specificity, 90 non-*Cronobacter* strains were tested and all produced no PCR products (Supplementary Table S4).

### Sensitivity of Duplex and Multiplex PCR Assay

**Figure 6:**
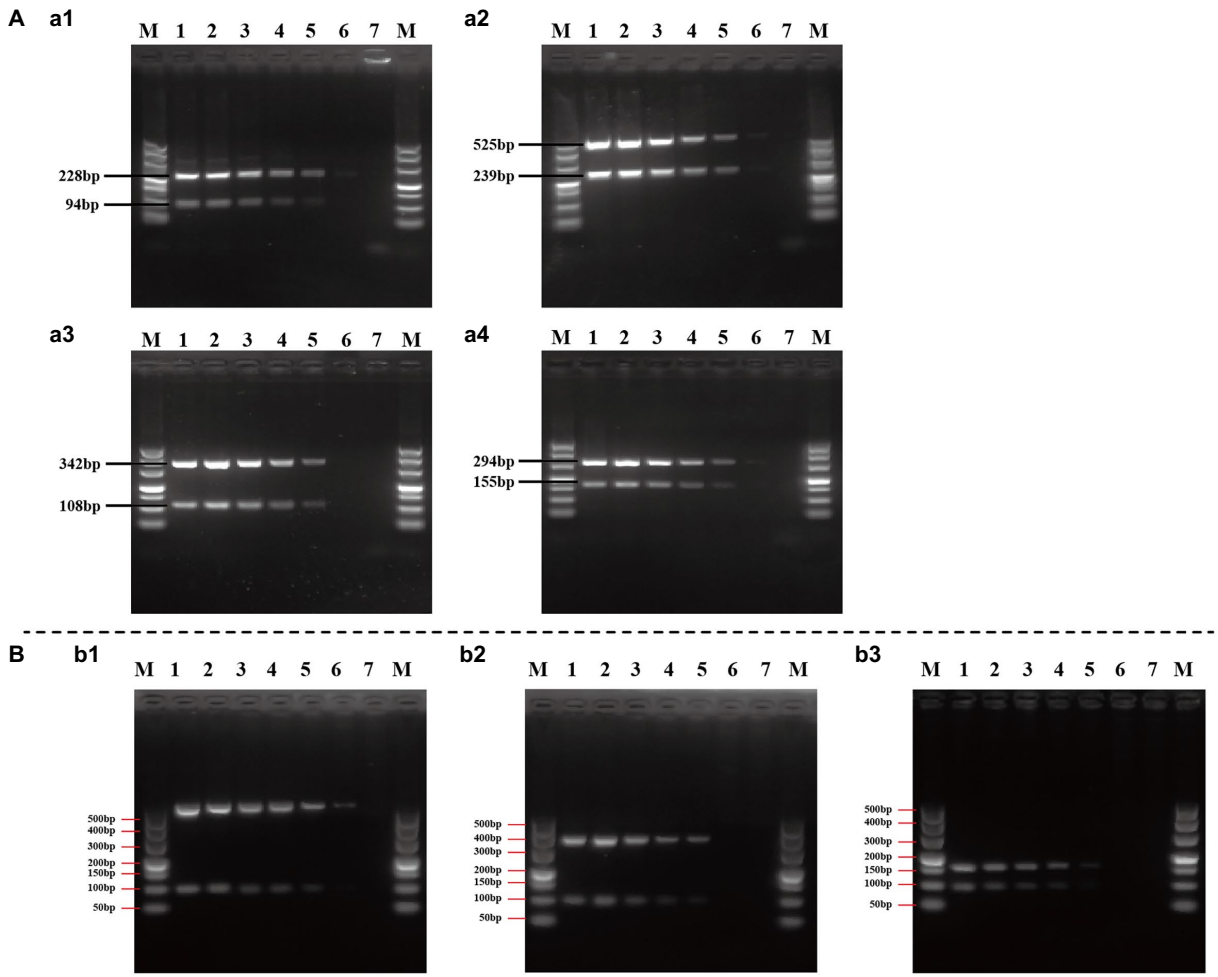
Evaluation of the sensitivity of duplex **(A)** and multiplex **(B)** PCR assay. **(a1–a4)**: *Cronobacter sakazakii* ATCC 29544™, *C. sakazakii* ATCC 29544™, *Cronobacter malonaticus* LC07, and *Cronobacter turicensis* LC08 as template to evaluate the sensitivity of duplex PCR assay based on Cro_set, Sak_set, Mal_set, and Tur_set; **(b1–b3)**: *C. sakazakii* ATCC 29544™, *C. malonaticus* LC07, and *C. turicensis* LC08 as template to evaluate the sensitivity of multiplex PCR assay based on Cro_set. Lane M: DNA marker; lanes 1–6: template of pure culture from 2.5 × 10^7^ to 2.5 × 10^2^ CFU/ml; and lane 7: negative control without template.

The sensitivity (limit of detection) of duplex and multiplex PCR assay for the identification of *Cronobacter* spp. was evaluated using a serial 10-fold dilution in the range of 2.5 × 10^7^–2.5 × 10^2^ CFU/ml of pure cultures. The representative PCR assay using pure cultures of *C. sakazakii* ATCC 29544™, *C. malonaticus* LC07, and *C. turicensis* LC08 is shown in Figure 6. Although results were unstable when using 2.5 × 10^2^ CFU/ml pure culture of isolates from different *Cronobacter* species, visible and clear amplicons (positive signals) were generated with ≥2.5 × 10^3^ CFU/ml pure culture for all PCR reactions, indicating the limit of detection of both duplex (Figure 6A) and multiplex (Figure 6B) PCR method that had high sensitivity.

## Discussion

*Cronobacter* has been isolated from various environments, including foods and clinical sources, and several disease cases have been associated with the ingestion of *Cronobacter*-contaminated foods, such as powdered milk formula (Kalyantanda et al., 2015). Detection of *Cronobacter* spp., especially *C. sakazakii*, *C. malonaticus*, and *C. turicensis*, has become increasingly important in food safety and clinical diagnosis.

Methods based on PCR have been widely used for detection of pathogens because they are simple and rapid compared with conventional culture-based methods (Petti, 2007). In recent decades, molecular methods based on genes, such as 16S *rDNA*, *MMS*, *fusA*, *rpoB*, *ompA*, *gyrB*, *cgcA*, etc., have been used for the identification of *Cronobacter* (Malorny and Wagner, 2005; Seo and Brackett, 2005; Derzelle et al., 2007; Stoop et al., 2009; Sonbol et al., 2013; Zimmermann et al., 2014; Li et al., 2017), however, only a few are used to differentiate *Cronobacter* at the species level. Therefore, it is essential to identify specific novel marker genes for *Cronobacter* spp. Owing to advancements in high-throughput sequencing technologies; it is possible to employ large-scale genome sequences for identification of highly specific marker genes. Goay et al. (2016) have identified five novel *Salmonella Typhi*-specific genes as markers for diagnosis of typhoid fever based on comparative genomic analysis; Hazen et al. (2016) have found several genes specific for lethal enteropathogenic *E. coli* using LS-BSR. Therefore, comparative genomic analysis would be practical to screen specific marker genes for rapid and precise detection of pathogens. Although Shang et al. (2021) obtained several *Cronobacter* species-specific genes through pan-genome analysis; screening threshold was based on 95% (not 100%) of the target genomes and 5% (not 0%) of the non-target genomes. Lee et al. (2022) identified 16 genes specific for *C. sakazakii*, but these genes were screened only from 17 genomes of *Cronobacter*. Therefore, the present study was the first approach to screen novel marker genes for both at genus and species level of *Cronobacter* based on large-scale genomic analysis from 799 genomes of *Cronobacter* and 136,146 genomes of non-*Cronobacter*. Thereafter, duplex and multiplex PCR methods were established according to these marker genes. Moreover, the specificity of duplex and multiplex PCR methods was validated with 74 *Cronobacter* and 90 non-*Cronobacter* strains. The results showed that *C. sakazakii*, *C. malonaticus*, and *C. turicensis* could be detected accurately at both the genus and species level, and *C. universalis* and *C. dublinensis* could be detected accurately at the genus level. However, it is difficult to evaluate the detection of *C. muytjensii* and *C. condimenti* due to the lack of strains. The sensitivity of duplex and multiplex PCR assay was also determined and the detection limit was 2.5 × 10^3^ CFU/ml for pure culture.

In summary, we successfully screened out *Cronobacter* genus- and species-specific marker genes using large-scale genomic analysis, and the specificity and sensitivity of these selected targets were evaluated using duplex and multiplex PCR. Thus, the established methods described here were proved to be reliable and sensitive for the identification of *Cronobacter* spp.

## Data Availability Statement

The datasets presented in this study can be found in online Cronobacter PubMLST database (https://pubmlst.org/organisms/cronobacter-spp/). The names of the repositories and accession numbers can be found in the **Supplementary Material**.

## Author Contributions

LW, MW, and BL designed the research. PW, YS, and YW performed the research. MW and XG provided technical support and insights. LW and LY analyzed the data. LW, XG, LY, and BL wrote the manuscript. All authors contributed to the article and approved the submitted version.

## Funding

This work was supported by National Natural Science Foundation of China (NSFC) Program (32070130 and 81772148), Young Scholar of Tianjin (20JCJQJC00180), the Committee on Science and Technology of Tianjin (19YFZCSN00080), and Health Commission of Hubei Province Foundation (WJ2019H528).

## Conflict of Interest

The authors declare that the research was conducted in the absence of any commercial or financial relationships that could be construed as a potential conflict of interest.

## Publisher’s Note

All claims expressed in this article are solely those of the authors and do not necessarily represent those of their affiliated organizations, or those of the publisher, the editors and the reviewers. Any product that may be evaluated in this article, or claim that may be made by its manufacturer, is not guaranteed or endorsed by the publisher.

## Supplementary Material

The Supplementary Material for this article can be found online at: https://www.frontiersin.org/articles/10.3389/fmicb.2022.885543/full#supplementary-material

Supplementary Figure S1 Design of PCR primers based on genus- and species-specific genes. Primer positions and directions are indicated as red arrows; genes lengths are indicated as black lines; variable and conserved (from magenta to purple) nucleotide positions are highlighted in the color stripe.

Supplementary Figure S2 Large-scale BLAST score ratio (LS-BSR) analysis of *Cronobacter sakazakii* specific marker genes *fimG* **(A)** and *nanK* **(B)**. **(a)** BSR value distribution of target marker genes in *C. sakazakii*, **(b)** BSR value distribution of target marker genes in the other six species of *Cronobacter*, and **(c)** BSR value distribution of target marker genes in other bacteria.

Supplementary Figure S3 Large-scale BLAST score ratio (LS-BSR) analysis of *C. malonatious* specific marker genes *papD* **(A)** and *sthD* **(B)**. **(a)** BSR value distribution of target marker genes in *Cronobacter malonaticus*, **(b)** BSR value distribution of target marker genes in the other six species of *Cronobacter*, and **(c)** BSR value distribution of target marker genes in other bacteria.

Supplementary Figure S4 Large-scale BLAST score ratio (LS-BSR) analysis of *C. turicensis* specific marker genes *phpB* **(A)** and *nudI* **(B)**. **(a)** BSR value distribution of target marker genes in *C. turicensis*, **(b)** BSR value distribution of target marker genes in the other six species of *Cronobacter*, and **(c)** BSR value distribution of target marker genes in other bacteria.

## References

[ref1] Carter L. Lindsey L. A. Grlm C. J. Sathymoorthy V. Jarvls K. G. Goplnath G.. (2013). Multiplex PCR assay targeting a diguanylate cyclase-encoding gene, *cgcA*, to differentiate species within the genus *Cronobacter*. Appl. Environ. Microbiol. 79, 734–737. doi: 10.1128/AEM.02898-12, PMID: 23144142 PMC3553758

[ref2] Derzelle S. Dilasser F. Maladen V. Soudrie N. Leclercq A. Lombard B.. (2007). Comparison of three chromogenic media and evaluation of two molecular-based identification systems for the detection of *Enterobacter sakazakii* from environmental samples from infant formulae factories. J. Food Prot. 70, 1678–1684. doi: 10.4315/0362-028X-70.7.1678, PMID: 17685342

[ref3] Drudy D. Mullane N. R. Quinn T. Wall P. G. Fanning S. (2006). *Enterobacter sakazakii*: an emerging pathogen in powdered infant formula. Clin. Infect. Dis. 42, 996–1002. doi: 10.1086/501019 16511766

[ref4] Emms D. M. Kelly S. (2015). OrthoFinder: solving fundamental biases in whole genome comparisons dramatically improves orthogroup inference accuracy. Genome Biol. 16:157. doi: 10.1186/s13059-015-0721-2, PMID: 26243257 PMC4531804

[ref5] Feeney A. Kropp K. A. O’connor R. Sleator R. D. (2014). *Cronobacter sakazakii*: stress survival and virulence potential in an opportunistic foodborne pathogen. Gut Microbes 5, 711–718. doi: 10.4161/19490976.2014.983774, PMID: 25562731 PMC4615781

[ref6] Forsythe S. J. (2018). Updates on the *Cronobacter* genus. Annu. Rev. Food Sci. Technol. 9, 23–44. doi: 10.1146/annurev-food-030117-012246 29272187

[ref7] Goay Y. X. Chin K. L. Tan C. L. L. Yeoh C. Y. Ja'afar J. A. N. Zaidah A. R.. (2016). Identification of five novel *Salmonella Typhi*-specific genes as markers for diagnosis of typhoid fever using single-gene target PCR assays. Biomed. Res. Int. 2016:8905675. doi: 10.1155/2016/8905675, PMID: 27975062 PMC5126401

[ref8] Hazen T. H. Donnenberg M. S. Panchalingam S. Antonio M. Hossain A. Mandomando I.. (2016). Genomic diversity of EPEC associated with clinical presentations of differing severity. Nat. Microbiol. 1:15014. doi: 10.1038/nmicrobiol.2015.14, PMID: 27571975 PMC5067155

[ref9] Huang C.-H. Chang M.-T. Huang L. (2013). Use of novel species-specific PCR primers targeted to DNA gyrase subunit B (*gyrB*) gene for species identification of the *Cronobacter* sakazakii and *Cronobacter dublinensis*. Mol. Cell. Probes 27, 15–18. doi: 10.1016/j.mcp.2012.08.004, PMID: 22963906

[ref10] Jain C. Rodriguez-R L. M. Phillippy A. M. Konstantinidis K. T. Aluru S. (2018). High throughput ANI analysis of 90K prokaryotic genomes reveals clear species boundaries. Nat. Commun. 9:5114. doi: 10.1038/s41467-018-07641-9, PMID: 30504855 PMC6269478

[ref11] Joseph S. Sonbol H. Hariri S. H. Desai P. T. Mcclelland M. Forsythe S. J. (2012). Diversity of the *Cronobacter* genus as revealed by multilocus sequence typing. J. Clin. Microbiol. 50, 3031–3039. doi: 10.1128/JCM.00905-12, PMID: 22785185 PMC3421776

[ref12] Kalyantanda G. Shumyak L. Archibald L. K. (2015). *Cronobacter* species contamination of powdered infant formula and the implications for neonatal health. Front. Pediatr. 3:56. doi: 10.3389/fped.2015.00056, PMID: 26191519 PMC4489094

[ref13] Katoh K. Standley D. M. (2013). MAFFT multiple sequence alignment software version 7: improvements in performance and usability. Mol. Biol. Evol. 30, 772–780. doi: 10.1093/molbev/mst010, PMID: 23329690 PMC3603318

[ref14] Killer J. Skrivanova E. Hochel I. Marounek M. (2015). Multilocus sequence typing of *Cronobacter* strains isolated from retail foods and environmental samples. Foodborne Pathog. Dis. 12, 514–521. doi: 10.1089/fpd.2014.1884, PMID: 25974656

[ref15] Lee J.-I. Kim S.-S. Kang D.-H. (2022). Development of DNA probes to detect *Cronobacter sakazakii* based on comparative genomics and its application in food samples. Food Control 137:108853. doi: 10.1016/j.foodcont.2022.108853

[ref16] Li X. Cui J. Du X. Cui Z. Huang Y. Kan B. (2017). Duplex real-time PCR method for the differentiation of *Cronobacter sakazakii* and *Cronobacter malonaticus*. J. Food Prot. 80, 50–56. doi: 10.4315/0362-028X.JFP-16-171, PMID: 28221877

[ref17] Li Y. Zhang L. Hu Y. Hong C. Xie A. Wu Y.. (2020). Prevalence and genetic characteristics of *Cronobacter* spp. from food and human clinical stool samples in Wenzhou, China 2008-2018. Food Microbiol. 89:103432. doi: 10.1016/j.fm.2020.103432, PMID: 32138990

[ref18] Ling N. Li C. Zhang J. Wu Q. Zeng H. He W.. (2018). Prevalence and molecular and antimicrobial characteristics of *Cronobacter* spp. isolated from raw vegetables in China. Front. Microbiol. 9:1149 doi: 10.3389/fmicb.2018.01149 29922254 PMC5996200

[ref19] Malorny B. Wagner M. (2005). Detection of *Enterobacter sakazakii* strains by real-time PCR. J. Food Prot. 68, 1623–1627. doi: 10.4315/0362-028X-68.8.1623 21132969

[ref20] Masood N. Moore K. Farbos A. Paszkiewicz K. Dickins B. Mcnally A.. (2015). Genomic dissection of the 1994 *Cronobacter sakazakii* outbreak in a French neonatal intensive care unit. BMC Genomics 16:750. doi: 10.1186/s12864-015-1961-y, PMID: 26438044 PMC4594962

[ref21] Petti C. A. (2007). Detection and identification of microorganisms by gene amplification and sequencing. Clin. Infect. Dis. 44, 1108–1114. doi: 10.1086/512818, PMID: 17366460

[ref22] Sahl J. W. Caporaso J. G. Rasko D. A. Keim P. (2014). The large-scale blast score ratio (LS-BSR) pipeline: a method to rapidly compare genetic content between bacterial genomes. PeerJ 2:e332. doi: 10.7717/peerj.332, PMID: 24749011 PMC3976120

[ref23] Seo K. H. Brackett R. E. (2005). Rapid, specific detection of *Enterobacter sakazakii* in infant formula using a real-time PCR assay. J. Food Prot. 68, 59–63. doi: 10.4315/0362-028X-68.1.59, PMID: 15690804

[ref24] Shang Y. Ye Q. Wu Q. Pang R. Zhou B. Wang C.. (2021). PCR and multiplex PCR assays for the detection of *Cronobacter* species using specific targets obtained by a bioinformatics approach. Food Control 125:107896. doi: 10.1016/j.foodcont.2021.107896

[ref25] Singh N. Goel G. Raghav M. (2015). Prevalence and characterization of *Cronobacter* spp. from various foods, medicinal plants, and environmental samples. Curr. Microbiol. 71, 31–38. doi: 10.1007/s00284-015-0816-8, PMID: 25855303

[ref26] Sonbol H. Joseph S. Mcauley C. M. Craven H. M. Forsythe S. J. (2013). Multilocus sequence typing of *Cronobacter* spp. from powdered infant formula and milk powder production factories. Int. Dairy J. 30, 1–7. doi: 10.1016/j.idairyj.2012.11.004

[ref27] Stamatakis A. (2014). RAxML version 8: a tool for phylogenetic analysis and post-analysis of large phylogenies. Bioinformatics 30, 1312–1313. doi: 10.1093/bioinformatics/btu033, PMID: 24451623 PMC3998144

[ref28] Stoop B. Lehner A. Iversen C. Fanning S. Stephan R. (2009). Development and evaluation of *rpoB* based PCR systems to differentiate the six proposed species within the genus *Cronobacter*. Int. J. Food Microbiol. 136, 165–168. doi: 10.1016/j.ijfoodmicro.2009.04.023, PMID: 19467725

[ref29] Yu G. Smith D. K. Zhu H. Guan Y. Lam T. T. Y. Mcinerny G. (2016). Ggtree: an r package for visualization and annotation of phylogenetic trees with their covariates and other associated data. Methods Ecol. Evol. 8, 28–36. doi: 10.1111/2041-210X.12628

[ref30] Zimmermann J. Schmidt H. Loessner M. J. Weiss A. (2014). Development of a rapid detection system for opportunistic pathogenic *Cronobacter* spp. in powdered milk products. Food Microbiol. 42, 19–25. doi: 10.1016/j.fm.2014.02.010, PMID: 24929712

