# Supplementary material for: Detection of Genus and Three Important Species of Cronobacter Using Novel Genus- and Species-Specific Genes Identified by Large-Scale Comparative Genomic Analysis
Source: Front Microbiol. 2022 Jun 2;13:885543. doi: 10.3389/fmicb.2022.885543 (PMC9201440; doi:10.3389/fmicb.2022.885543)
Supplement: Supplementary file 2 [file Table_1.DOCX]

**Supplementary Materials**

**Supplementary Table S1.** Strains utilized for *in vitro* validation.

| **strain** | **species** | **source#** | **strain** | **species** | **source** |
| --- | --- | --- | --- | --- | --- |
| 29544 | *Cronobacter sakazakii* | ATCC^TM^ | LC41 | *Cronobacter sakazakii* | IQCC |
| BAA-894 | *Cronobacter sakazakii* | ATCC^TM^ | LC42 | *Cronobacter sakazakii* | CICC |
| LC01 | *Cronobacter dublinensis* | CCM | LC43 | *Cronobacter sakazakii* | IQCC |
| LC02 | *Cronobacter dublinensis* | IQCC | LC44 | *Cronobacter sakazakii* | IQCC |
| LC04 | *Cronobacter dublinensis* | BEEIQB | LC45 | *Cronobacter sakazakii* | IQCC |
| LC05 | *Cronobacter dublinensis* | BEEIQB | LC46 | *Cronobacter sakazakii* | IQCC |
| LC13 | *Cronobacter dublinensis* | IQCC | LC47 | *Cronobacter sakazakii* | BEEIQB |
| LC03 | *Cronobacter malonaticus* | IQCC | LC48 | *Cronobacter sakazakii* | BEEIQB |
| LC07 | *Cronobacter malonaticus* | IQCC | LC49 | *Cronobacter sakazakii* | BEEIQB |
| LC11 | *Cronobacter malonaticus* | CICC | LC50 | *Cronobacter sakazakii* | BEEIQB |
| LC06 | *Cronobacter sakazakii* | TEEIQB | LC51 | *Cronobacter sakazakii* | BEEIQB |
| LC15 | *Cronobacter sakazakii* | ATCC^TM^ | LC52 | *Cronobacter sakazakii* | BEEIQB |
| LC16 | *Cronobacter sakazakii* | TEEIQB | LC53 | *Cronobacter sakazakii* | BEEIQB |
| LC17 | *Cronobacter sakazakii* | ATCC^TM^ | LC54 | *Cronobacter sakazakii* | BEEIQB |
| LC18 | *Cronobacter sakazakii* | CCM | LC55 | *Cronobacter sakazakii* | BEEIQB |
| LC19 | *Cronobacter sakazakii* | CICC | LC56 | *Cronobacter sakazakii* | BEEIQB |
| LC20 | *Cronobacter sakazakii* | CICC | LC57 | *Cronobacter sakazakii* | BEEIQB |
| LC21 | *Cronobacter sakazakii* | IQCC | LC58 | *Cronobacter sakazakii* | BEEIQB |
| LC22 | *Cronobacter sakazakii* | IQCC | LC59 | *Cronobacter sakazakii* | BEEIQB |
| LC23 | *Cronobacter sakazakii* | IQCC | LC60 | *Cronobacter sakazakii* | BEEIQB |
| LC24 | *Cronobacter sakazakii* | IQCC | LC61 | *Cronobacter sakazakii* | TEEIQB |
| LC25 | *Cronobacter sakazakii* | IQCC | LC62 | *Cronobacter sakazakii* | TEEIQB |
| LC26 | *Cronobacter sakazakii* | IQCC | LC63 | *Cronobacter sakazakii* | TEEIQB |
| LC27 | *Cronobacter sakazakii* | BEEIQB | LC64 | *Cronobacter sakazakii* | TEEIQB |
| LC28 | *Cronobacter sakazakii* | BEEIQB | LC65 | *Cronobacter sakazakii* | TEEIQB |
| LC29 | *Cronobacter sakazakii* | BEEIQB | LC66 | *Cronobacter sakazakii* | TEEIQB |
| LC30 | *Cronobacter sakazakii* | BEEIQB | LC67 | *Cronobacter sakazakii* | TEEIQB |
| LC31 | *Cronobacter sakazakii* | TEEIQB | LC68 | *Cronobacter sakazakii* | TEEIQB |
| LC32 | *Cronobacter sakazakii* | TEEIQB | LC69 | *Cronobacter sakazakii* | BEEIQB |
| LC33 | *Cronobacter sakazakii* | TEEIQB | LC70 | *Cronobacter sakazakii* | BEEIQB |
| LC34 | *Cronobacter sakazakii* | TEEIQB | LC71 | *Cronobacter sakazakii* | BEEIQB |
| LC35 | *Cronobacter sakazakii* | TEEIQB | LC72 | *Cronobacter sakazakii* | TEEIQB |
| LC36 | *Cronobacter sakazakii* | IQCC | LC73 | *Cronobacter sakazakii* | TEEIQB |
| LC37 | *Cronobacter sakazakii* | IQCC | LC08 | *Cronobacter turicensis* | CICC |
| LC38 | *Cronobacter sakazakii* | IQCC | LC12 | *Cronobacter turicensis* | IQCC |
| LC39 | *Cronobacter sakazakii* | IQCC | LC09 | *Cronobacter universalis* | CICC |
| LC40 | *Cronobacter sakazakii* | IQCC | LC10 | *Cronobacter universalis* | CICC |
| AS 1.181 | *Enterobacter cloacae* | CGMCC | eae006 | *Enterobacter aerogenes* | SCDC |
| AS 1.1733 | *Enterobacter cloacae* | CGMCC | eae007 | *Enterobacter aerogenes* | SCDC |
| 45301-6 | *Enterobacter cloacae* | CMCC | eae008 | *Enterobacter aerogenes* | SCDC |
| 10523 | *Enterobacter cloacae* | ACCC | eae009 | *Enterobacter aerogenes* | SCDC |
| 13047 | *Enterobacter cloacae* | ATCC^TM^ | eae010 | *Enterobacter aerogenes* | SCDC |
| 1.57 | *Enterobacter cloacae* | CGMCC | eae011 | *Enterobacter aerogenes* | SCDC |
| 1.58 | *Enterobacter cloacae* | CGMCC | eae012 | *Enterobacter aerogenes* | SCDC |
| 1.242 | *Enterobacter cloacae* | CGMCC | eae013 | *Enterobacter aerogenes* | SCDC |
| 1.243 | *Enterobacter cloacae* | CGMCC | eae014 | *Enterobacter aerogenes* | SCDC |
| 10014 | *Enterobacter cloacae* | CICC | JM109 | *Escherichia coli* | Lab |
| 10017 | *Enterobacter cloacae* | CICC | DH5a | *Escherichia coli* | Lab |
| 1.58 | *Enterobacter cloacae* | CGMCC | BL21 | *Escherichia coli* | Lab |
| 1.242 | *Enterobacter cloacae* | CGMCC | EDL933 | *Escherichia coli* | ATCC^TM^ |
| 1.243 | *Enterobacter cloacae* | CGMCC | 44274 | *Escherichia coli* | CMCC |
| 1.1015 | *Enterobacter cloacae* | CGMCC | CB9767 | *Escherichia coli* | BfR |
| 1.1016 | *Enterobacter cloacae* | CGMCC | C1003-63 | *Escherichia coli* | BfR |
| 1.57 | *Enterobacter cloacae* | CGMCC | CB176 | *Escherichia coli* | BfR |
| 1.2022 | *Enterobacter cloacae* | CGMCC | 315-60 | *Escherichia coli* | BfR |
| 51697 | *Enterobacter aerogenes* | ATCC^TM^ | CB9720 | *Escherichia coli* | BfR |
| 35029 | *Enterobacter aerogenes* | ATCC^TM^ | CB9723 | *Escherichia coli* | BfR |
| 29007 | *Enterobacter aerogenes* | ATCC^TM^ | C339-54 | *Escherichia coli* | BfR |
| 29940 | *Enterobacter aerogenes* | ATCC^TM^ | 637-62 | *Escherichia coli* | BfR |
| 15038 | *Enterobacter aerogenes* | ATCC^TM^ | 4155 | *Escherichia coli* | BfR |
| 13048 | *Enterobacter aerogenes* | ATCC^TM^ | CB9421 | *Escherichia coli* | BfR |
| 29010 | *Enterobacter aerogenes* | ATCC^TM^ | CB9752 | *Escherichia coli* | BfR |
| 56257 | *Enterobacter aerogenes* | SCDC | 26W | *Escherichia coli* | BfR |
| 54429 | *Enterobacter aerogenes* | SCDC | 556-59 | *Escherichia coli* | BfR |
| 54316 | *Enterobacter aerogenes* | SCDC | 240-58 | *Escherichia coli* | BfR |
| 55961 | *Enterobacter aerogenes* | SCDC | 339-54 | *Escherichia coli* | BfR |
| 55615 | *Enterobacter aerogenes* | SCDC | CB9468 | *Escherichia coli* | BfR |
| 55751 | *Enterobacter aerogenes* | SCDC | C3813 | *Escherichia coli* | BfR |
| 55147 | *Enterobacter aerogenes* | SCDC | C288-63 | *Escherichia coli* | BfR |
| 56362 | *Enterobacter aerogenes* | SCDC | C275-53 | *Escherichia coli* | BfR |
| 55734 | *Enterobacter aerogenes* | SCDC | 539-83 | *Escherichia coli* | BfR |
| 54605 | *Enterobacter aerogenes* | SCDC | F7902-41 | *Escherichia coli* | BfR |
| 55889 | *Enterobacter aerogenes* | SCDC | M1466 | *Escherichia coli* | SMMB |
| 54133 | *Enterobacter aerogenes* | SCDC | M1465 | *Escherichia coli* | SMMB |
| 54063 | *Enterobacter aerogenes* | SCDC | M1459 | *Escherichia coli* | SMMB |
| 54191 | *Enterobacter aerogenes* | SCDC | M1484 | *Escherichia coli* | SMMB |
| 54696 | *Enterobacter aerogenes* | SCDC | M1473 | *Escherichia coli* | SMMB |
| eae001 | *Enterobacter aerogenes* | SCDC | M1474 | *Escherichia coli* | SMMB |
| eae002 | *Enterobacter aerogenes* | SCDC | M1475 | *Escherichia coli* | SMMB |
| eae003 | *Enterobacter aerogenes* | SCDC | M1261 | *Escherichia coli* | SMMB |
| eae004 | *Enterobacter aerogenes* | SCDC | M1478 | *Escherichia coli* | SMMB |
| eae005 | *Enterobacter aerogenes* | SCDC | M1485 | *Escherichia coli* | SMMB |

^#^ BEEIQB: isolates from food obtained at the Beijing Entry-Exit Inspection and Quarantine Bureau, China.

TEEIQB: isolates from food obtained at the Tianjin Entry-Exit Inspection and Quarantine Bureau, China

SCDC: Shanghai Center For Disease Control And Prevention

CCM: Czech Collection of Microorganisms

ATCC: American Type Culture Collection

IQCC: Inspection & Quarantine Culture Collection, Chinese Academy of Inspection and Quarantine

CICC: China Center of Industrial Culture Collection

CGMCC: China General Microbiological Culture Collection Center

CMCC: China Medical Culture Collection Center

BfR: Berlin Federal Institute for Risk Assessment (BfR)

SMMB: School of Molecular and Microbial Biosciences, University of Sydney

ACCC: Agricultural Culture Collection of China

Lab: Strain stored in our laboratory

**Supplementary Table S2.** Genomes of *Cronobacter* utilized in this study.

| **no.** | **id^a^** | **species** | **no.** | **id** | **species** | **no.** | **id** | **species** |
| --- | --- | --- | --- | --- | --- | --- | --- | --- |
| 1 | id1098 | *C. sakazakii* | 268 | id2421 | *C. sakazakii* | 535 | id3105 | *C. sakazakii* |
| 2 | id1099 | *C. sakazakii* | 269 | id2422 | *C. sakazakii* | 536 | id3106 | *C. sakazakii* |
| 3 | id1100 | *C. sakazakii* | 270 | id2423 | *C. sakazakii* | 537 | id3107 | *C. sakazakii* |
| 4 | id1101 | *C. sakazakii* | 271 | id2424 | *C. sakazakii* | 538 | id3108 | *C. sakazakii* |
| 5 | id1102 | *C. sakazakii* | 272 | id2425 | *C. sakazakii* | 539 | id3109 | *C. sakazakii* |
| 6 | id1103 | *C. sakazakii* | 273 | id2426 | *C. sakazakii* | 540 | id3110 | *C. sakazakii* |
| 7 | id1104 | *C. sakazakii* | 274 | id2427 | *C. sakazakii* | 541 | id3111 | *C. sakazakii* |
| 8 | id1105 | *C. sakazakii* | 275 | id2428 | *C. sakazakii* | 542 | id3112 | *C. sakazakii* |
| 9 | id1107 | *C. sakazakii* | 276 | id2429 | *C. sakazakii* | 543 | id3122 | *C. sakazakii* |
| 10 | id1108 | *C. sakazakii* | 277 | id2430 | *C. sakazakii* | 544 | id3123 | *C. sakazakii* |
| 11 | id1109 | *C. sakazakii* | 278 | id2431 | *C. sakazakii* | 545 | id3124 | *C. sakazakii* |
| 12 | id1110 | *C. sakazakii* | 279 | id2432 | *C. sakazakii* | 546 | id3125 | *C. sakazakii* |
| 13 | id1111 | *C. sakazakii* | 280 | id2433 | *C. sakazakii* | 547 | id3126 | *C. sakazakii* |
| 14 | id1112 | *C. sakazakii* | 281 | id2434 | *C. sakazakii* | 548 | id3127 | *C. sakazakii* |
| 15 | id1113 | *C. sakazakii* | 282 | id2435 | *C. sakazakii* | 549 | id3128 | *C. sakazakii* |
| 16 | id1114 | *C. sakazakii* | 283 | id2436 | *C. sakazakii* | 550 | id3129 | *C. sakazakii* |
| 17 | id1115 | *C. sakazakii* | 284 | id2437 | *C. sakazakii* | 551 | id3130 | *C. sakazakii* |
| 18 | id1116 | *C. sakazakii* | 285 | id2438 | *C. sakazakii* | 552 | id3131 | *C. sakazakii* |
| 19 | id1117 | *C. sakazakii* | 286 | id2439 | *C. sakazakii* | 553 | id3132 | *C. sakazakii* |
| 20 | id1118 | *C. sakazakii* | 287 | id2440 | *C. sakazakii* | 554 | id355 | *C. sakazakii* |
| 21 | id1119 | *C. sakazakii* | 288 | id2441 | *C. sakazakii* | 555 | id420 | *C. sakazakii* |
| 22 | id1120 | *C. sakazakii* | 289 | id2442 | *C. sakazakii* | 556 | id421 | *C. sakazakii* |
| 23 | id1121 | *C. sakazakii* | 290 | id2443 | *C. sakazakii* | 557 | id425 | *C. sakazakii* |
| 24 | id1122 | *C. sakazakii* | 291 | id2444 | *C. sakazakii* | 558 | id426 | *C. sakazakii* |
| 25 | id1123 | *C. sakazakii* | 292 | id2445 | *C. sakazakii* | 559 | id428 | *C. sakazakii* |
| 26 | id1124 | *C. sakazakii* | 293 | id2456 | *C. sakazakii* | 560 | id429 | *C. sakazakii* |
| 27 | id1126 | *C. sakazakii* | 294 | id2475 | *C. sakazakii* | 561 | id430 | *C. sakazakii* |
| 28 | id1127 | *C. sakazakii* | 295 | id2477 | *C. sakazakii* | 562 | id431 | *C. sakazakii* |
| 29 | id1130 | *C. sakazakii* | 296 | id2478 | *C. sakazakii* | 563 | id432 | *C. sakazakii* |
| 30 | id1134 | *C. sakazakii* | 297 | id2479 | *C. sakazakii* | 564 | id433 | *C. sakazakii* |
| 31 | id1135 | *C. sakazakii* | 298 | id2480 | *C. sakazakii* | 565 | id436 | *C. sakazakii* |
| 32 | id1136 | *C. sakazakii* | 299 | id2481 | *C. sakazakii* | 566 | id446 | *C. sakazakii* |
| 33 | id1137 | *C. sakazakii* | 300 | id2482 | *C. sakazakii* | 567 | id46 | *C. sakazakii* |
| 34 | id1138 | *C. sakazakii* | 301 | id2483 | *C. sakazakii* | 568 | id5 | *C. sakazakii* |
| 35 | id1139 | *C. sakazakii* | 302 | id2484 | *C. sakazakii* | 569 | id50 | *C. sakazakii* |
| 36 | id1140 | *C. sakazakii* | 303 | id2485 | *C. sakazakii* | 570 | id57 | *C. sakazakii* |
| 37 | id1141 | *C. sakazakii* | 304 | id2486 | *C. sakazakii* | 571 | id591 | *C. sakazakii* |
| 38 | id1142 | *C. sakazakii* | 305 | id2487 | *C. sakazakii* | 572 | id592 | *C. sakazakii* |
| 39 | id1143 | *C. sakazakii* | 306 | id2488 | *C. sakazakii* | 573 | id593 | *C. sakazakii* |
| 40 | id1144 | *C. sakazakii* | 307 | id2495 | *C. sakazakii* | 574 | id594 | *C. sakazakii* |
| 41 | id1145 | *C. sakazakii* | 308 | id2496 | *C. sakazakii* | 575 | id653 | *C. sakazakii* |
| 42 | id1146 | *C. sakazakii* | 309 | id2497 | *C. sakazakii* | 576 | id654 | *C. sakazakii* |
| 43 | id1147 | *C. sakazakii* | 310 | id2498 | *C. sakazakii* | 577 | id848 | *C. sakazakii* |
| 44 | id1148 | *C. sakazakii* | 311 | id2499 | *C. sakazakii* | 578 | id923 | *C. sakazakii* |
| 45 | id1149 | *C. sakazakii* | 312 | id2500 | *C. sakazakii* | 579 | id1129 | *C. malonaticus* |
| 46 | id1150 | *C. sakazakii* | 313 | id2501 | *C. sakazakii* | 580 | id1132 | *C. malonaticus* |
| 47 | id1151 | *C. sakazakii* | 314 | id2503 | *C. sakazakii* | 581 | id1133 | *C. malonaticus* |
| 48 | id1152 | *C. sakazakii* | 315 | id2504 | *C. sakazakii* | 582 | id1170 | *C. malonaticus* |
| 49 | id1153 | *C. sakazakii* | 316 | id2505 | *C. sakazakii* | 583 | id1174 | *C. malonaticus* |
| 50 | id1154 | *C. sakazakii* | 317 | id2506 | *C. sakazakii* | 584 | id1176 | *C. malonaticus* |
| 51 | id1155 | *C. sakazakii* | 318 | id2507 | *C. sakazakii* | 585 | id1177 | *C. malonaticus* |
| 52 | id1158 | *C. sakazakii* | 319 | id2508 | *C. sakazakii* | 586 | id1185 | *C. malonaticus* |
| 53 | id1159 | *C. sakazakii* | 320 | id2509 | *C. sakazakii* | 587 | id1186 | *C. malonaticus* |
| 54 | id1160 | *C. sakazakii* | 321 | id2510 | *C. sakazakii* | 588 | id1190 | *C. malonaticus* |
| 55 | id1161 | *C. sakazakii* | 322 | id2511 | *C. sakazakii* | 589 | id129 | *C. malonaticus* |
| 56 | id1162 | *C. sakazakii* | 323 | id2512 | *C. sakazakii* | 590 | id1313 | *C. malonaticus* |
| 57 | id1163 | *C. sakazakii* | 324 | id2513 | *C. sakazakii* | 591 | id133 | *C. malonaticus* |
| 58 | id1164 | *C. sakazakii* | 325 | id2514 | *C. sakazakii* | 592 | id144 | *C. malonaticus* |
| 59 | id1187 | *C. sakazakii* | 326 | id2515 | *C. sakazakii* | 593 | id145 | *C. malonaticus* |
| 60 | id1188 | *C. sakazakii* | 327 | id2516 | *C. sakazakii* | 594 | id1479 | *C. malonaticus* |
| 61 | id1189 | *C. sakazakii* | 328 | id2517 | *C. sakazakii* | 595 | id1484 | *C. malonaticus* |
| 62 | id120 | *C. sakazakii* | 329 | id2518 | *C. sakazakii* | 596 | id1485 | *C. malonaticus* |
| 63 | id121 | *C. sakazakii* | 330 | id2519 | *C. sakazakii* | 597 | id1488 | *C. malonaticus* |
| 64 | id1314 | *C. sakazakii* | 331 | id2520 | *C. sakazakii* | 598 | id1489 | *C. malonaticus* |
| 65 | id1315 | *C. sakazakii* | 332 | id2521 | *C. sakazakii* | 599 | id1491 | *C. malonaticus* |
| 66 | id1316 | *C. sakazakii* | 333 | id2522 | *C. sakazakii* | 600 | id1494 | *C. malonaticus* |
| 67 | id1317 | *C. sakazakii* | 334 | id2523 | *C. sakazakii* | 601 | id1499 | *C. malonaticus* |
| 68 | id1318 | *C. sakazakii* | 335 | id2524 | *C. sakazakii* | 602 | id1503 | *C. malonaticus* |
| 69 | id1319 | *C. sakazakii* | 336 | id2525 | *C. sakazakii* | 603 | id1556 | *C. malonaticus* |
| 70 | id1320 | *C. sakazakii* | 337 | id2526 | *C. sakazakii* | 604 | id1759 | *C. malonaticus* |
| 71 | id1321 | *C. sakazakii* | 338 | id2527 | *C. sakazakii* | 605 | id176 | *C. malonaticus* |
| 72 | id1322 | *C. sakazakii* | 339 | id2528 | *C. sakazakii* | 606 | id1760 | *C. malonaticus* |
| 73 | id1324 | *C. sakazakii* | 340 | id2529 | *C. sakazakii* | 607 | id1763 | *C. malonaticus* |
| 74 | id1325 | *C. sakazakii* | 341 | id2530 | *C. sakazakii* | 608 | id177 | *C. malonaticus* |
| 75 | id1327 | *C. sakazakii* | 342 | id2531 | *C. sakazakii* | 609 | id1806 | *C. malonaticus* |
| 76 | id1328 | *C. sakazakii* | 343 | id2532 | *C. sakazakii* | 610 | id1842 | *C. malonaticus* |
| 77 | id1329 | *C. sakazakii* | 344 | id2533 | *C. sakazakii* | 611 | id1843 | *C. malonaticus* |
| 78 | id1332 | *C. sakazakii* | 345 | id2534 | *C. sakazakii* | 612 | id1844 | *C. malonaticus* |
| 79 | id1336 | *C. sakazakii* | 346 | id2535 | *C. sakazakii* | 613 | id185 | *C. malonaticus* |
| 80 | id1337 | *C. sakazakii* | 347 | id2536 | *C. sakazakii* | 614 | id2004 | *C. malonaticus* |
| 81 | id1338 | *C. sakazakii* | 348 | id2537 | *C. sakazakii* | 615 | id213 | *C. malonaticus* |
| 82 | id1339 | *C. sakazakii* | 349 | id2538 | *C. sakazakii* | 616 | id2250 | *C. malonaticus* |
| 83 | id1340 | *C. sakazakii* | 350 | id2539 | *C. sakazakii* | 617 | id2489 | *C. malonaticus* |
| 84 | id1344 | *C. sakazakii* | 351 | id2540 | *C. sakazakii* | 618 | id2554 | *C. malonaticus* |
| 85 | id1345 | *C. sakazakii* | 352 | id2541 | *C. sakazakii* | 619 | id2555 | *C. malonaticus* |
| 86 | id1346 | *C. sakazakii* | 353 | id2542 | *C. sakazakii* | 620 | id2556 | *C. malonaticus* |
| 87 | id1347 | *C. sakazakii* | 354 | id2543 | *C. sakazakii* | 621 | id2557 | *C. malonaticus* |
| 88 | id1348 | *C. sakazakii* | 355 | id2544 | *C. sakazakii* | 622 | id2558 | *C. malonaticus* |
| 89 | id1350 | *C. sakazakii* | 356 | id2545 | *C. sakazakii* | 623 | id2559 | *C. malonaticus* |
| 90 | id1352 | *C. sakazakii* | 357 | id2546 | *C. sakazakii* | 624 | id2560 | *C. malonaticus* |
| 91 | id1353 | *C. sakazakii* | 358 | id2547 | *C. sakazakii* | 625 | id2561 | *C. malonaticus* |
| 92 | id1354 | *C. sakazakii* | 359 | id2548 | *C. sakazakii* | 626 | id2562 | *C. malonaticus* |
| 93 | id1355 | *C. sakazakii* | 360 | id2549 | *C. sakazakii* | 627 | id2563 | *C. malonaticus* |
| 94 | id1358 | *C. sakazakii* | 361 | id2550 | *C. sakazakii* | 628 | id2567 | *C. malonaticus* |
| 95 | id1359 | *C. sakazakii* | 362 | id2551 | *C. sakazakii* | 629 | id2568 | *C. malonaticus* |
| 96 | id1361 | *C. sakazakii* | 363 | id2552 | *C. sakazakii* | 630 | id2569 | *C. malonaticus* |
| 97 | id1363 | *C. sakazakii* | 364 | id2573 | *C. sakazakii* | 631 | id2571 | *C. malonaticus* |
| 98 | id1364 | *C. sakazakii* | 365 | id2574 | *C. sakazakii* | 632 | id2572 | *C. malonaticus* |
| 99 | id1365 | *C. sakazakii* | 366 | id2575 | *C. sakazakii* | 633 | id2639 | *C. malonaticus* |
| 100 | id1366 | *C. sakazakii* | 367 | id2576 | *C. sakazakii* | 634 | id2640 | *C. malonaticus* |
| 101 | id1367 | *C. sakazakii* | 368 | id2577 | *C. sakazakii* | 635 | id2642 | *C. malonaticus* |
| 102 | id1368 | *C. sakazakii* | 369 | id2578 | *C. sakazakii* | 636 | id266 | *C. malonaticus* |
| 103 | id1369 | *C. sakazakii* | 370 | id2579 | *C. sakazakii* | 637 | id2687 | *C. malonaticus* |
| 104 | id1372 | *C. sakazakii* | 371 | id2580 | *C. sakazakii* | 638 | id2697 | *C. malonaticus* |
| 105 | id1386 | *C. sakazakii* | 372 | id2581 | *C. sakazakii* | 639 | id2713 | *C. malonaticus* |
| 106 | id147 | *C. sakazakii* | 373 | id2583 | *C. sakazakii* | 640 | id2715 | *C. malonaticus* |
| 107 | id1478 | *C. sakazakii* | 374 | id2584 | *C. sakazakii* | 641 | id2718 | *C. malonaticus* |
| 108 | id1482 | *C. sakazakii* | 375 | id2585 | *C. sakazakii* | 642 | id2956 | *C. malonaticus* |
| 109 | id1483 | *C. sakazakii* | 376 | id2586 | *C. sakazakii* | 643 | id2962 | *C. malonaticus* |
| 110 | id1486 | *C. sakazakii* | 377 | id2587 | *C. sakazakii* | 644 | id2966 | *C. malonaticus* |
| 111 | id1487 | *C. sakazakii* | 378 | id2588 | *C. sakazakii* | 645 | id2968 | *C. malonaticus* |
| 112 | id1493 | *C. sakazakii* | 379 | id2589 | *C. sakazakii* | 646 | id2969 | *C. malonaticus* |
| 113 | id1495 | *C. sakazakii* | 380 | id2590 | *C. sakazakii* | 647 | id2978 | *C. malonaticus* |
| 114 | id1502 | *C. sakazakii* | 381 | id2591 | *C. sakazakii* | 648 | id3031 | *C. malonaticus* |
| 115 | id1505 | *C. sakazakii* | 382 | id2592 | *C. sakazakii* | 649 | id3079 | *C. malonaticus* |
| 116 | id1506 | *C. sakazakii* | 383 | id2593 | *C. sakazakii* | 650 | id3113 | *C. malonaticus* |
| 117 | id154 | *C. sakazakii* | 384 | id2594 | *C. sakazakii* | 651 | id3114 | *C. malonaticus* |
| 118 | id1558 | *C. sakazakii* | 385 | id2595 | *C. sakazakii* | 652 | id3115 | *C. malonaticus* |
| 119 | id1673 | *C. sakazakii* | 386 | id2596 | *C. sakazakii* | 653 | id3116 | *C. malonaticus* |
| 120 | id1674 | *C. sakazakii* | 387 | id2597 | *C. sakazakii* | 654 | id3117 | *C. malonaticus* |
| 121 | id1751 | *C. sakazakii* | 388 | id2598 | *C. sakazakii* | 655 | id3118 | *C. malonaticus* |
| 122 | id1752 | *C. sakazakii* | 389 | id2599 | *C. sakazakii* | 656 | id3119 | *C. malonaticus* |
| 123 | id1753 | *C. sakazakii* | 390 | id2600 | *C. sakazakii* | 657 | id3120 | *C. malonaticus* |
| 124 | id1754 | *C. sakazakii* | 391 | id2601 | *C. sakazakii* | 658 | id3121 | *C. malonaticus* |
| 125 | id1755 | *C. sakazakii* | 392 | id2602 | *C. sakazakii* | 659 | id316 | *C. malonaticus* |
| 126 | id1756 | *C. sakazakii* | 393 | id2603 | *C. sakazakii* | 660 | id385 | *C. malonaticus* |
| 127 | id1757 | *C. sakazakii* | 394 | id2604 | *C. sakazakii* | 661 | id399 | *C. malonaticus* |
| 128 | id1758 | *C. sakazakii* | 395 | id2605 | *C. sakazakii* | 662 | id400 | *C. malonaticus* |
| 129 | id1761 | *C. sakazakii* | 396 | id2606 | *C. sakazakii* | 663 | id402 | *C. malonaticus* |
| 130 | id1762 | *C. sakazakii* | 397 | id2607 | *C. sakazakii* | 664 | id405 | *C. malonaticus* |
| 131 | id1833 | *C. sakazakii* | 398 | id2608 | *C. sakazakii* | 665 | id406 | *C. malonaticus* |
| 132 | id1834 | *C. sakazakii* | 399 | id2609 | *C. sakazakii* | 666 | id407 | *C. malonaticus* |
| 133 | id1835 | *C. sakazakii* | 400 | id2610 | *C. sakazakii* | 667 | id415 | *C. malonaticus* |
| 134 | id1836 | *C. sakazakii* | 401 | id2611 | *C. sakazakii* | 668 | id418 | *C. malonaticus* |
| 135 | id1837 | *C. sakazakii* | 402 | id2612 | *C. sakazakii* | 669 | id434 | *C. malonaticus* |
| 136 | id1838 | *C. sakazakii* | 403 | id2613 | *C. sakazakii* | 670 | id590 | *C. malonaticus* |
| 137 | id1839 | *C. sakazakii* | 404 | id2614 | *C. sakazakii* | 671 | id61 | *C. malonaticus* |
| 138 | id1840 | *C. sakazakii* | 405 | id2615 | *C. sakazakii* | 672 | id619 | *C. malonaticus* |
| 139 | id186 | *C. sakazakii* | 406 | id2616 | *C. sakazakii* | 673 | id620 | *C. malonaticus* |
| 140 | id1897 | *C. sakazakii* | 407 | id2617 | *C. sakazakii* | 674 | id655 | *C. malonaticus* |
| 141 | id1898 | *C. sakazakii* | 408 | id2635 | *C. sakazakii* | 675 | id71 | *C. malonaticus* |
| 142 | id1899 | *C. sakazakii* | 409 | id2637 | *C. sakazakii* | 676 | id74 | *C. malonaticus* |
| 143 | id1900 | *C. sakazakii* | 410 | id2638 | *C. sakazakii* | 677 | id75 | *C. malonaticus* |
| 144 | id1901 | *C. sakazakii* | 411 | id2641 | *C. sakazakii* | 678 | id76 | *C. malonaticus* |
| 145 | id1902 | *C. sakazakii* | 412 | id2643 | *C. sakazakii* | 679 | id110 | *C. turicensis* |
| 146 | id1903 | *C. sakazakii* | 413 | id2644 | *C. sakazakii* | 680 | id1125 | *C. turicensis* |
| 147 | id1904 | *C. sakazakii* | 414 | id2645 | *C. sakazakii* | 681 | id1171 | *C. turicensis* |
| 148 | id1905 | *C. sakazakii* | 415 | id2654 | *C. sakazakii* | 682 | id1172 | *C. turicensis* |
| 149 | id1906 | *C. sakazakii* | 416 | id2681 | *C. sakazakii* | 683 | id1178 | *C. turicensis* |
| 150 | id1907 | *C. sakazakii* | 417 | id2682 | *C. sakazakii* | 684 | id1331 | *C. turicensis* |
| 151 | id1908 | *C. sakazakii* | 418 | id2683 | *C. sakazakii* | 685 | id1349 | *C. turicensis* |
| 152 | id1909 | *C. sakazakii* | 419 | id2684 | *C. sakazakii* | 686 | id1370 | *C. turicensis* |
| 153 | id1910 | *C. sakazakii* | 420 | id2685 | *C. sakazakii* | 687 | id1371 | *C. turicensis* |
| 154 | id1911 | *C. sakazakii* | 421 | id2686 | *C. sakazakii* | 688 | id1490 | *C. turicensis* |
| 155 | id1912 | *C. sakazakii* | 422 | id2688 | *C. sakazakii* | 689 | id1501 | *C. turicensis* |
| 156 | id1913 | *C. sakazakii* | 423 | id2689 | *C. sakazakii* | 690 | id1713 | *C. turicensis* |
| 157 | id1914 | *C. sakazakii* | 424 | id2690 | *C. sakazakii* | 691 | id1845 | *C. turicensis* |
| 158 | id1915 | *C. sakazakii* | 425 | id2691 | *C. sakazakii* | 692 | id1932 | *C. turicensis* |
| 159 | id1916 | *C. sakazakii* | 426 | id2692 | *C. sakazakii* | 693 | id2235 | *C. turicensis* |
| 160 | id1917 | *C. sakazakii* | 427 | id2693 | *C. sakazakii* | 694 | id2249 | *C. turicensis* |
| 161 | id1918 | *C. sakazakii* | 428 | id2694 | *C. sakazakii* | 695 | id2394 | *C. turicensis* |
| 162 | id1919 | *C. sakazakii* | 429 | id2695 | *C. sakazakii* | 696 | id2564 | *C. turicensis* |
| 163 | id1920 | *C. sakazakii* | 430 | id2696 | *C. sakazakii* | 697 | id2565 | *C. turicensis* |
| 164 | id1921 | *C. sakazakii* | 431 | id2698 | *C. sakazakii* | 698 | id2825 | *C. turicensis* |
| 165 | id1922 | *C. sakazakii* | 432 | id2699 | *C. sakazakii* | 699 | id2826 | *C. turicensis* |
| 166 | id1923 | *C. sakazakii* | 433 | id270 | *C. sakazakii* | 700 | id2827 | *C. turicensis* |
| 167 | id1924 | *C. sakazakii* | 434 | id2700 | *C. sakazakii* | 701 | id2979 | *C. turicensis* |
| 168 | id1925 | *C. sakazakii* | 435 | id2701 | *C. sakazakii* | 702 | id2982 | *C. turicensis* |
| 169 | id1926 | *C. sakazakii* | 436 | id2702 | *C. sakazakii* | 703 | id2996 | *C. turicensis* |
| 170 | id1927 | *C. sakazakii* | 437 | id2703 | *C. sakazakii* | 704 | id2999 | *C. turicensis* |
| 171 | id1928 | *C. sakazakii* | 438 | id2704 | *C. sakazakii* | 705 | id3003 | *C. turicensis* |
| 172 | id1929 | *C. sakazakii* | 439 | id2705 | *C. sakazakii* | 706 | id3010 | *C. turicensis* |
| 173 | id1930 | *C. sakazakii* | 440 | id2706 | *C. sakazakii* | 707 | id3011 | *C. turicensis* |
| 174 | id1931 | *C. sakazakii* | 441 | id2707 | *C. sakazakii* | 708 | id3012 | *C. turicensis* |
| 175 | id1933 | *C. sakazakii* | 442 | id2709 | *C. sakazakii* | 709 | id3013 | *C. turicensis* |
| 176 | id1934 | *C. sakazakii* | 443 | id2710 | *C. sakazakii* | 710 | id3014 | *C. turicensis* |
| 177 | id1935 | *C. sakazakii* | 444 | id2711 | *C. sakazakii* | 711 | id3077 | *C. turicensis* |
| 178 | id1936 | *C. sakazakii* | 445 | id2712 | *C. sakazakii* | 712 | id435 | *C. turicensis* |
| 179 | id1937 | *C. sakazakii* | 446 | id2714 | *C. sakazakii* | 713 | id82 | *C. turicensis* |
| 180 | id1938 | *C. sakazakii* | 447 | id2716 | *C. sakazakii* | 714 | id1554 | *C. condimenti* |
| 181 | id1939 | *C. sakazakii* | 448 | id2717 | *C. sakazakii* | 715 | id1896 | *C. condimenti* |
| 182 | id1940 | *C. sakazakii* | 449 | id2719 | *C. sakazakii* | 716 | id1131 | *C. dublinensis* |
| 183 | id1941 | *C. sakazakii* | 450 | id2720 | *C. sakazakii* | 717 | id1173 | *C. dublinensis* |
| 184 | id1942 | *C. sakazakii* | 451 | id2721 | *C. sakazakii* | 718 | id1175 | *C. dublinensis* |
| 185 | id1943 | *C. sakazakii* | 452 | id2723 | *C. sakazakii* | 719 | id1184 | *C. dublinensis* |
| 186 | id1944 | *C. sakazakii* | 453 | id2724 | *C. sakazakii* | 720 | id1323 | *C. dublinensis* |
| 187 | id1945 | *C. sakazakii* | 454 | id2803 | *C. sakazakii* | 721 | id1326 | *C. dublinensis* |
| 188 | id1946 | *C. sakazakii* | 455 | id2804 | *C. sakazakii* | 722 | id1330 | *C. dublinensis* |
| 189 | id1947 | *C. sakazakii* | 456 | id2849 | *C. sakazakii* | 723 | id1333 | *C. dublinensis* |
| 190 | id1948 | *C. sakazakii* | 457 | id2851 | *C. sakazakii* | 724 | id1334 | *C. dublinensis* |
| 191 | id1949 | *C. sakazakii* | 458 | id2852 | *C. sakazakii* | 725 | id1335 | *C. dublinensis* |
| 192 | id1950 | *C. sakazakii* | 459 | id2955 | *C. sakazakii* | 726 | id1341 | *C. dublinensis* |
| 193 | id1951 | *C. sakazakii* | 460 | id2957 | *C. sakazakii* | 727 | id1342 | *C. dublinensis* |
| 194 | id1952 | *C. sakazakii* | 461 | id2958 | *C. sakazakii* | 728 | id1343 | *C. dublinensis* |
| 195 | id1953 | *C. sakazakii* | 462 | id2959 | *C. sakazakii* | 729 | id1351 | *C. dublinensis* |
| 196 | id1954 | *C. sakazakii* | 463 | id2960 | *C. sakazakii* | 730 | id1356 | *C. dublinensis* |
| 197 | id1955 | *C. sakazakii* | 464 | id2961 | *C. sakazakii* | 731 | id1357 | *C. dublinensis* |
| 198 | id1956 | *C. sakazakii* | 465 | id2963 | *C. sakazakii* | 732 | id1360 | *C. dublinensis* |
| 199 | id1957 | *C. sakazakii* | 466 | id2964 | *C. sakazakii* | 733 | id1362 | *C. dublinensis* |
| 200 | id1958 | *C. sakazakii* | 467 | id2965 | *C. sakazakii* | 734 | id140 | *C. dublinensis* |
| 201 | id1959 | *C. sakazakii* | 468 | id2970 | *C. sakazakii* | 735 | id146 | *C. dublinensis* |
| 202 | id1960 | *C. sakazakii* | 469 | id2971 | *C. sakazakii* | 736 | id148 | *C. dublinensis* |
| 203 | id1961 | *C. sakazakii* | 470 | id2976 | *C. sakazakii* | 737 | id1492 | *C. dublinensis* |
| 204 | id1962 | *C. sakazakii* | 471 | id2980 | *C. sakazakii* | 738 | id151 | *C. dublinensis* |
| 205 | id1963 | *C. sakazakii* | 472 | id2981 | *C. sakazakii* | 739 | id152 | *C. dublinensis* |
| 206 | id1964 | *C. sakazakii* | 473 | id2983 | *C. sakazakii* | 740 | id153 | *C. dublinensis* |
| 207 | id1965 | *C. sakazakii* | 474 | id2994 | *C. sakazakii* | 741 | id1555 | *C. dublinensis* |
| 208 | id1966 | *C. sakazakii* | 475 | id2997 | *C. sakazakii* | 742 | id1679 | *C. dublinensis* |
| 209 | id1967 | *C. sakazakii* | 476 | id3000 | *C. sakazakii* | 743 | id1683 | *C. dublinensis* |
| 210 | id1968 | *C. sakazakii* | 477 | id3001 | *C. sakazakii* | 744 | id1684 | *C. dublinensis* |
| 211 | id1969 | *C. sakazakii* | 478 | id3002 | *C. sakazakii* | 745 | id1686 | *C. dublinensis* |
| 212 | id1970 | *C. sakazakii* | 479 | id3004 | *C. sakazakii* | 746 | id1709 | *C. dublinensis* |
| 213 | id1971 | *C. sakazakii* | 480 | id3005 | *C. sakazakii* | 747 | id1717 | *C. dublinensis* |
| 214 | id1972 | *C. sakazakii* | 481 | id3006 | *C. sakazakii* | 748 | id1721 | *C. dublinensis* |
| 215 | id1973 | *C. sakazakii* | 482 | id3007 | *C. sakazakii* | 749 | id1748 | *C. dublinensis* |
| 216 | id1974 | *C. sakazakii* | 483 | id3008 | *C. sakazakii* | 750 | id1856 | *C. dublinensis* |
| 217 | id1975 | *C. sakazakii* | 484 | id3018 | *C. sakazakii* | 751 | id1871 | *C. dublinensis* |
| 218 | id1976 | *C. sakazakii* | 485 | id3019 | *C. sakazakii* | 752 | id1873 | *C. dublinensis* |
| 219 | id1977 | *C. sakazakii* | 486 | id3020 | *C. sakazakii* | 753 | id192 | *C. dublinensis* |
| 220 | id1978 | *C. sakazakii* | 487 | id3021 | *C. sakazakii* | 754 | id193 | *C. dublinensis* |
| 221 | id1979 | *C. sakazakii* | 488 | id3022 | *C. sakazakii* | 755 | id194 | *C. dublinensis* |
| 222 | id1980 | *C. sakazakii* | 489 | id3023 | *C. sakazakii* | 756 | id196 | *C. dublinensis* |
| 223 | id1981 | *C. sakazakii* | 490 | id3024 | *C. sakazakii* | 757 | id197 | *C. dublinensis* |
| 224 | id1982 | *C. sakazakii* | 491 | id3025 | *C. sakazakii* | 758 | id2001 | *C. dublinensis* |
| 225 | id1983 | *C. sakazakii* | 492 | id3026 | *C. sakazakii* | 759 | id2490 | *C. dublinensis* |
| 226 | id1984 | *C. sakazakii* | 493 | id3027 | *C. sakazakii* | 760 | id2491 | *C. dublinensis* |
| 227 | id1985 | *C. sakazakii* | 494 | id3028 | *C. sakazakii* | 761 | id2492 | *C. dublinensis* |
| 228 | id1986 | *C. sakazakii* | 495 | id3029 | *C. sakazakii* | 762 | id2493 | *C. dublinensis* |
| 229 | id1987 | *C. sakazakii* | 496 | id3030 | *C. sakazakii* | 763 | id2494 | *C. dublinensis* |
| 230 | id1988 | *C. sakazakii* | 497 | id3035 | *C. sakazakii* | 764 | id2566 | *C. dublinensis* |
| 231 | id1989 | *C. sakazakii* | 498 | id3040 | *C. sakazakii* | 765 | id2824 | *C. dublinensis* |
| 232 | id1990 | *C. sakazakii* | 499 | id3041 | *C. sakazakii* | 766 | id2954 | *C. dublinensis* |
| 233 | id1991 | *C. sakazakii* | 500 | id3044 | *C. sakazakii* | 767 | id2967 | *C. dublinensis* |
| 234 | id1992 | *C. sakazakii* | 501 | id3045 | *C. sakazakii* | 768 | id2972 | *C. dublinensis* |
| 235 | id1993 | *C. sakazakii* | 502 | id3046 | *C. sakazakii* | 769 | id2974 | *C. dublinensis* |
| 236 | id1994 | *C. sakazakii* | 503 | id3047 | *C. sakazakii* | 770 | id2977 | *C. dublinensis* |
| 237 | id1995 | *C. sakazakii* | 504 | id3050 | *C. sakazakii* | 771 | id2998 | *C. dublinensis* |
| 238 | id1996 | *C. sakazakii* | 505 | id3051 | *C. sakazakii* | 772 | id443 | *C. dublinensis* |
| 239 | id1997 | *C. sakazakii* | 506 | id3052 | *C. sakazakii* | 773 | id595 | *C. dublinensis* |
| 240 | id1998 | *C. sakazakii* | 507 | id3053 | *C. sakazakii* | 774 | id596 | *C. dublinensis* |
| 241 | id1999 | *C. sakazakii* | 508 | id3071 | *C. sakazakii* | 775 | id597 | *C. dublinensis* |
| 242 | id2000 | *C. sakazakii* | 509 | id3072 | *C. sakazakii* | 776 | id124 | *C. muytjensii* |
| 243 | id2098 | *C. sakazakii* | 510 | id3076 | *C. sakazakii* | 777 | id126 | *C. muytjensii* |
| 244 | id2240 | *C. sakazakii* | 511 | id3080 | *C. sakazakii* | 778 | id1497 | *C. muytjensii* |
| 245 | id2241 | *C. sakazakii* | 512 | id3081 | *C. sakazakii* | 779 | id1498 | *C. muytjensii* |
| 246 | id2242 | *C. sakazakii* | 513 | id3082 | *C. sakazakii* | 780 | id1504 | *C. muytjensii* |
| 247 | id2243 | *C. sakazakii* | 514 | id3083 | *C. sakazakii* | 781 | id1559 | *C. muytjensii* |
| 248 | id2244 | *C. sakazakii* | 515 | id3084 | *C. sakazakii* | 782 | id1841 | *C. muytjensii* |
| 249 | id2245 | *C. sakazakii* | 516 | id3085 | *C. sakazakii* | 783 | id187 | *C. muytjensii* |
| 250 | id2246 | *C. sakazakii* | 517 | id3086 | *C. sakazakii* | 784 | id200 | *C. muytjensii* |
| 251 | id2247 | *C. sakazakii* | 518 | id3087 | *C. sakazakii* | 785 | id2973 | *C. muytjensii* |
| 252 | id2252 | *C. sakazakii* | 519 | id3088 | *C. sakazakii* | 786 | id3075 | *C. muytjensii* |
| 253 | id2253 | *C. sakazakii* | 520 | id3089 | *C. sakazakii* | 787 | id3104 | *C. muytjensii* |
| 254 | id2254 | *C. sakazakii* | 521 | id3090 | *C. sakazakii* | 788 | id442 | *C. muytjensii* |
| 255 | id2255 | *C. sakazakii* | 522 | id3091 | *C. sakazakii* | 789 | id598 | *C. muytjensii* |
| 256 | id2256 | *C. sakazakii* | 523 | id3092 | *C. sakazakii* | 790 | id935 | *C. muytjensii* |
| 257 | id2257 | *C. sakazakii* | 524 | id3093 | *C. sakazakii* | 791 | id1477 | *C. universalis* |
| 258 | id2258 | *C. sakazakii* | 525 | id3094 | *C. sakazakii* | 792 | id1496 | *C. universalis* |
| 259 | id2393 | *C. sakazakii* | 526 | id3095 | *C. sakazakii* | 793 | id1507 | *C. universalis* |
| 260 | id2395 | *C. sakazakii* | 527 | id3096 | *C. sakazakii* | 794 | id1557 | *C. universalis* |
| 261 | id2396 | *C. sakazakii* | 528 | id3097 | *C. sakazakii* | 795 | id156 | *C. universalis* |
| 262 | id2397 | *C. sakazakii* | 529 | id3098 | *C. sakazakii* | 796 | id2570 | *C. universalis* |
| 263 | id24 | *C. sakazakii* | 530 | id3099 | *C. sakazakii* | 797 | id408 | *C. universalis* |
| 264 | id2417 | *C. sakazakii* | 531 | id3100 | *C. sakazakii* | 798 | id599 | *C. universalis* |
| 265 | id2418 | *C. sakazakii* | 532 | id3101 | *C. sakazakii* | 799 | id84 | *C. universalis* |
| 266 | id2419 | *C. sakazakii* | 533 | id3102 | *C. sakazakii* |  |  |  |
| 267 | id2420 | *C. sakazakii* | 534 | id3103 | *C. sakazakii* |  |  |  |
| **^a^** genome id from *Cronobacter* PubMLST database. | | | | | | | | |

**Supplementary Table S3.** Genomes and relative taxonomic position of non-*Cronobacter* analyzed in this study.

| **Phylum** | **Class** | **Order** | **Family** | **Genus** | **Species** | **Genomes^a^** |
| --- | --- | --- | --- | --- | --- | --- |
| Actinobacteria | actinobacteria | Corynebacteriales | *Mycobacteriaceae* | *Mycobacterium* | *M. tuberculosis* | 5478 |
|  |  |  |  | *Mycobacteroides* | *M. abscessus* | 1425 |
| Firmicutes | Bacilli | Bacillales | *Bacillaceae* | *Bacillus* | *B. cereus* | 997 |
|  |  |  |  |  | *B. subtilis* | 290 |
|  |  |  | *Listeriaceae* | *Listeria* | *L. booriae* | 60 |
|  |  |  |  |  | *L. innocua* | 141 |
|  |  |  |  |  | *L. ivanovii* | 31 |
|  |  |  |  |  | *L. marthii* | 17 |
|  |  |  |  |  | *L. monocytogenes* | 19892 |
|  |  |  |  |  | *L. seeligeri* | 126 |
|  |  |  | *Staphylococcaceae* | *Staphylococcus* | *S. aureus* | 418 |
|  |  |  |  |  | *S. epidermidis* | 1987 |
|  |  |  |  |  | *S. haemolyticus* | 276 |
|  |  |  |  |  | *S. pseudintermedius* | 1568 |
|  |  |  | *Micrococcaceae* | *Micrococcus* | *M. luteus* | 46 |
|  |  | Lactobacillales | *streptococcaceae* | *Streptococcus* | *S. agalactiae* | 1383 |
|  |  |  |  |  | *S. pneumoniae* | 7066 |
|  |  |  |  |  | *S. pyogenes* | 2183 |
|  |  |  | *Enterococcaceae* | *Enterococcus* | *E. faecalis* | 3492 |
|  |  |  |  |  | *E. faecium* | 7953 |
|  | Cloastridia | Clostridiales | *Peptostreptococcaceae* | *Clostridioides* | *C. difficile* | 3239 |
|  |  |  | *Clostridium* | *Clostridium* | *C. botulinum* | 690 |
|  |  |  |  |  | *C. perfringens* | 326 |
| Proteobacteria | α-proteobacteria | Rhizobiales | *Rhizobiaceae* | *Agrobacterium* | *A. rhizogenes* | 56 |
|  |  |  |  |  | *A. vitis* | 24 |
|  |  |  |  | *Rhizobium* | *R. leguminosarum* | 350 |
|  |  |  |  |  | *R. phaseoli* | 11 |
|  |  |  |  | *Bradyrhizobium* | *B. japonicum* | 17 |
|  |  |  | *Brucellaceae* | *Brucella* | *B. abortus* | 211 |
|  |  |  |  |  | *B. melitensis* | 278 |
|  | β-proteobacteria | Neisseriales | *Neisseriaceae* | *Neisseria* | *N. gonorrhoeae* | 628 |
|  |  |  |  |  | *N. meningitidis* | 2615 |
|  | γ-proteobacteria | Enterobacterales | *Enterobacteriaceae* | *Escherichia* | *E. albertii* | 50 |
|  |  |  |  |  | *E. coli* | 10604 |
|  |  |  |  |  | *E. fergusonii* | 10 |
|  |  |  |  |  | *E. marmotae* | 2 |
|  |  |  |  | *Klebsiella* | *K. aerogenes* | 286 |
|  |  |  |  |  | *K. grimontii* | 105 |
|  |  |  |  |  | *K. huaxiensis* | 4 |
|  |  |  |  |  | *K. michiganensis* | 196 |
|  |  |  |  |  | *K. oxytoca* | 164 |
|  |  |  |  |  | *K. pneumoniae* | 10982 |
|  |  |  |  |  | *K. quasipneumoniae* | 496 |
|  |  |  |  |  | *K. quasivariicola* | 18 |
|  |  |  |  | *Enterobacter* | *E. asburiae* | 145 |
|  |  |  |  |  | *E. bugandensis* | 63 |
|  |  |  |  |  | *E. cancerogenus* | 16 |
|  |  |  |  |  | *E. chengduensis* | 9 |
|  |  |  |  |  | *E. chuandaensis* | 1 |
|  |  |  |  |  | *E. cloacae* | 803 |
|  |  |  |  |  | *E. hormaechei* | 1482 |
|  |  |  |  | *Citrobacter* | *C. amalonaticus* | 31 |
|  |  |  |  |  | *C. braakii* | 30 |
|  |  |  |  |  | *C. freundii* | 326 |
|  |  |  |  | *Shigella* | *S. boydii* | 94 |
|  |  |  |  |  | *S. dysenteriae* | 47 |
|  |  |  |  |  | *S. flexneri* | 223 |
|  |  |  |  |  | *S. sonnei* | 1052 |
|  |  |  |  | *Salmonella* | *S. bongori* | 12 |
|  |  |  |  |  | *S. enterica* | 8165 |
|  |  |  |  | *Siccibacter* | *Siccibacter* spp. | 8 |
|  |  |  |  | *Franconibacter* | *Franconibacter* spp. | 10 |
|  |  |  | *Yersiniaceae* | *Yersinia* | *Y. enterocolitica* | 388 |
|  |  |  |  |  | *Y. pestis* | 570 |
|  |  |  |  |  | *Y. pseudotuberculosis* | 298 |
|  |  |  |  |  | *Y. ruckeri* | 66 |
|  |  |  |  | *Serratia* | *S. marcescens* | 917 |
|  |  | Pseudomonadales | *pseudomonadaceae* | *Pseudomonas* | *P. aeruginosa* | 5903 |
|  |  |  |  |  | *P. fluorescens* | 176 |
|  |  |  |  |  | *P. putida* | 113 |
|  |  |  |  |  | *P. syringae* | 421 |
|  |  |  | *Moraxellaceae* | *Acinetobacter* | *A. baumannii* | 6907 |
|  |  |  |  |  | *A. bereziniae* | 49 |
|  |  | Vibrionales | *Vibrionaceae* | *Vibro* | *V. cholerae* | 800 |
|  |  |  |  |  | *V. parahaemolyticus* | 1410 |
|  |  | Pasteurellales | *pasteurellaceae* | *Haemophilus* | *H. haemolyticus* | 60 |
|  |  |  |  |  | *H. influenzae* | 752 |
|  |  |  |  |  | *H. parainfluenzae* | 63 |
|  |  | Legionellales | *Legionellaceae* | *Legionella* | *L. pneumophila* | 2615 |
|  | ε-proteobacteria | Campylobacterales | *Campylobacteraceae* | *Campylobacter* | *C. coli* | 14968 |
|  |  |  |  |  | *C. concisus* | 233 |
|  |  |  |  |  | *C. fetus* | 214 |
|  |  |  |  |  | *C. jejuni* | 515 |
| **^a^** the number of genomes of non-*Cronobacter* analyzed in this study. | | | | | | |

| **Supplementary Table S4.** Evaluation results of different primer sets for the 74 *Cronobacter* and 90 non-*Cronobacter* strains. | | | | | | |
| --- | --- | --- | --- | --- | --- | --- |
| **Strain** | **Species** | **Duplex PCR** | | | | **Multiplex PCR** |
|  |  | **Cro_set** | **Sak_set** | **Mal_set** | **Tur_set** | **CroM_set** |
| LC01 | *Cronobacter dublinensis* | **+^a^** | **-** | **-** | **-** | **+** |
| LC02 | *Cronobacter dublinensis* | **+** | **-** | **-** | **-** | **+** |
| LC04 | *Cronobacter dublinensis* | **+** | **-** | **-** | **-** | **+** |
| LC05 | *Cronobacter dublinensis* | **+** | **-** | **-** | **-** | **+** |
| LC13 | *Cronobacter dublinensis* | **+** | **-** | **-** | **-** | **+** |
| LC03 | *Cronobacter malonaticus* | **+** | **-** | **+** | **-** | **+** |
| LC07 | *Cronobacter malonaticus* | **+** | **-** | **+** | **-** | **+** |
| LC11 | *Cronobacter malonaticus* | **+** | **-** | **+** | **-** | **+** |
| 29544 | *Cronobacter sakazakii* | **+** | **+** | **-** | **-** | **+** |
| BAA-894 | *Cronobacter sakazakii* | **+** | **+** | **-** | **-** | **+** |
| LC06 | *Cronobacter sakazakii* | **+** | **+** | **-** | **-** | **+** |
| LC15 | *Cronobacter sakazakii* | **+** | **+** | **-** | **-** | **+** |
| LC16 | *Cronobacter sakazakii* | **+** | **+** | **-** | **-** | **+** |
| LC17 | *Cronobacter sakazakii* | **+** | **+** | **-** | **-** | **+** |
| LC18 | *Cronobacter sakazakii* | **+** | **+** | **-** | **-** | **+** |
| LC19 | *Cronobacter sakazakii* | **+** | **+** | **-** | **-** | **+** |
| LC20 | *Cronobacter sakazakii* | **+** | **+** | **-** | **-** | **+** |
| LC21 | *Cronobacter sakazakii* | **+** | **+** | **-** | **-** | **+** |
| LC22 | *Cronobacter sakazakii* | **+** | **+** | **-** | **-** | **+** |
| LC23 | *Cronobacter sakazakii* | **+** | **+** | **-** | **-** | **+** |
| LC24 | *Cronobacter sakazakii* | **+** | **+** | **-** | **-** | **+** |
| LC25 | *Cronobacter sakazakii* | **+** | **+** | **-** | **-** | **+** |
| LC26 | *Cronobacter sakazakii* | **+** | **+** | **-** | **-** | **+** |
| LC27 | *Cronobacter sakazakii* | **+** | **+** | **-** | **-** | **+** |
| LC28 | *Cronobacter sakazakii* | **+** | **+** | **-** | **-** | **+** |
| LC29 | *Cronobacter sakazakii* | **+** | **+** | **-** | **-** | **+** |
| LC30 | *Cronobacter sakazakii* | **+** | **+** | **-** | **-** | **+** |
| LC31 | *Cronobacter sakazakii* | **+** | **+** | **-** | **-** | **+** |
| LC32 | *Cronobacter sakazakii* | **+** | **+** | **-** | **-** | **+** |
| LC33 | *Cronobacter sakazakii* | **+** | **+** | **-** | **-** | **+** |
| LC34 | *Cronobacter sakazakii* | **+** | **+** | **-** | **-** | **+** |
| LC35 | *Cronobacter sakazakii* | **+** | **+** | **-** | **-** | **+** |
| LC36 | *Cronobacter sakazakii* | **+** | **+** | **-** | **-** | **+** |
| LC37 | *Cronobacter sakazakii* | **+** | **+** | **-** | **-** | **+** |
| LC38 | *Cronobacter sakazakii* | **+** | **+** | **-** | **-** | **+** |
| LC39 | *Cronobacter sakazakii* | **+** | **+** | **-** | **-** | **+** |
| LC40 | *Cronobacter sakazakii* | **+** | **+** | **-** | **-** | **+** |
| LC41 | *Cronobacter sakazakii* | **+** | **+** | **-** | **-** | **+** |
| LC42 | *Cronobacter sakazakii* | **+** | **+** | **-** | **-** | **+** |
| LC43 | *Cronobacter sakazakii* | **+** | **+** | **-** | **-** | **+** |
| LC44 | *Cronobacter sakazakii* | **+** | **+** | **-** | **-** | **+** |
| LC45 | *Cronobacter sakazakii* | **+** | **+** | **-** | **-** | **+** |
| LC46 | *Cronobacter sakazakii* | **+** | **+** | **-** | **-** | **+** |
| LC47 | *Cronobacter sakazakii* | **+** | **+** | **-** | **-** | **+** |
| LC48 | *Cronobacter sakazakii* | **+** | **+** | **-** | **-** | **+** |
| LC49 | *Cronobacter sakazakii* | **+** | **+** | **-** | **-** | **+** |
| LC50 | *Cronobacter sakazakii* | **+** | **+** | **-** | **-** | **+** |
| LC51 | *Cronobacter sakazakii* | **+** | **+** | **-** | **-** | **+** |
| LC52 | *Cronobacter sakazakii* | **+** | **+** | **-** | **-** | **+** |
| LC53 | *Cronobacter sakazakii* | **+** | **+** | **-** | **-** | **+** |
| LC54 | *Cronobacter sakazakii* | **+** | **+** | **-** | **-** | **+** |
| LC55 | *Cronobacter sakazakii* | **+** | **+** | **-** | **-** | **+** |
| LC56 | *Cronobacter sakazakii* | **+** | **+** | **-** | **-** | **+** |
| LC57 | *Cronobacter sakazakii* | **+** | **+** | **-** | **-** | **+** |
| LC58 | *Cronobacter sakazakii* | **+** | **+** | **-** | **-** | **+** |
| LC59 | *Cronobacter sakazakii* | **+** | **+** | **-** | **-** | **+** |
| LC60 | *Cronobacter sakazakii* | **+** | **+** | **-** | **-** | **+** |
| LC61 | *Cronobacter sakazakii* | **+** | **+** | **-** | **-** | **+** |
| LC62 | *Cronobacter sakazakii* | **+** | **+** | **-** | **-** | **+** |
| LC63 | *Cronobacter sakazakii* | **+** | **+** | **-** | **-** | **+** |
| LC64 | *Cronobacter sakazakii* | **+** | **+** | **-** | **-** | **+** |
| LC65 | *Cronobacter sakazakii* | **+** | **+** | **-** | **-** | **+** |
| LC66 | *Cronobacter sakazakii* | **+** | **+** | **-** | **-** | **+** |
| LC67 | *Cronobacter sakazakii* | **+** | **+** | **-** | **-** | **+** |
| LC68 | *Cronobacter sakazakii* | **+** | **+** | **-** | **-** | **+** |
| LC69 | *Cronobacter sakazakii* | **+** | **+** | **-** | **-** | **+** |
| LC70 | *Cronobacter sakazakii* | **+** | **+** | **-** | **-** | **+** |
| LC71 | *Cronobacter sakazakii* | **+** | **+** | **-** | **-** | **+** |
| LC72 | *Cronobacter sakazakii* | **+** | **+** | **-** | **-** | **+** |
| LC73 | *Cronobacter sakazakii* | **+** | **+** | **-** | **-** | **+** |
| LC08 | *Cronobacter turicensis* | **+** | **-** | **-** | **+** | **+** |
| LC12 | *Cronobacter turicensis* | **+** | **-** | **-** | **+** | **+** |
| LC09 | *Cronobacter universalis* | **+** | **-** | **-** | **-** | **+** |
| LC10 | *Cronobacter universalis* | **+** | **-** | **-** | **-** | **+** |
| AS 1.181 | *Enterobacter cloacae* | **-** | **-** | **-** | **-** | **-** |
| AS 1.1733 | *Enterobacter cloacae* | **-** | **-** | **-** | **-** | **-** |
| 45301-6 | *Enterobacter cloacae* | **-** | **-** | **-** | **-** | **-** |
| 10523 | *Enterobacter cloacae* | **-** | **-** | **-** | **-** | **-** |
| 13047 | *Enterobacter cloacae* | **-** | **-** | **-** | **-** | **-** |
| 1.57 | *Enterobacter cloacae* | **-** | **-** | **-** | **-** | **-** |
| 1.58 | *Enterobacter cloacae* | **-** | **-** | **-** | **-** | **-** |
| 1.242 | *Enterobacter cloacae* | **-** | **-** | **-** | **-** | **-** |
| 1.243 | *Enterobacter cloacae* | **-** | **-** | **-** | **-** | **-** |
| 10014 | *Enterobacter cloacae* | **-** | **-** | **-** | **-** | **-** |
| 10017 | *Enterobacter cloacae* | **-** | **-** | **-** | **-** | **-** |
| 1.58 | *Enterobacter cloacae* | **-** | **-** | **-** | **-** | **-** |
| 1.242 | *Enterobacter cloacae* | **-** | **-** | **-** | **-** | **-** |
| 1.243 | *Enterobacter cloacae* | **-** | **-** | **-** | **-** | **-** |
| 1.1015 | *Enterobacter cloacae* | **-** | **-** | **-** | **-** | **-** |
| 1.1016 | *Enterobacter cloacae* | **-** | **-** | **-** | **-** | **-** |
| 1.57 | *Enterobacter cloacae* | **-** | **-** | **-** | **-** | **-** |
| 1.2022 | *Enterobacter cloacae* | **-** | **-** | **-** | **-** | **-** |
| 51697 | *Enterobacter aerogenes* | **-** | **-** | **-** | **-** | **-** |
| 35029 | *Enterobacter aerogenes* | **-** | **-** | **-** | **-** | **-** |
| 29007 | *Enterobacter aerogenes* | **-** | **-** | **-** | **-** | **-** |
| 29940 | *Enterobacter aerogenes* | **-** | **-** | **-** | **-** | **-** |
| 15038 | *Enterobacter aerogenes* | **-** | **-** | **-** | **-** | **-** |
| 13048 | *Enterobacter aerogenes* | **-** | **-** | **-** | **-** | **-** |
| 29010 | *Enterobacter aerogenes* | **-** | **-** | **-** | **-** | **-** |
| 56257 | *Enterobacter aerogenes* | **-** | **-** | **-** | **-** | **-** |
| 54429 | *Enterobacter aerogenes* | **-** | **-** | **-** | **-** | **-** |
| 54316 | *Enterobacter aerogenes* | **-** | **-** | **-** | **-** | **-** |
| 55961 | *Enterobacter aerogenes* | **-** | **-** | **-** | **-** | **-** |
| 55615 | *Enterobacter aerogenes* | **-** | **-** | **-** | **-** | **-** |
| 55751 | *Enterobacter aerogenes* | **-** | **-** | **-** | **-** | **-** |
| 55147 | *Enterobacter aerogenes* | **-** | **-** | **-** | **-** | **-** |
| 56362 | *Enterobacter aerogenes* | **-** | **-** | **-** | **-** | **-** |
| 55734 | *Enterobacter aerogenes* | **-** | **-** | **-** | **-** | **-** |
| 54605 | *Enterobacter aerogenes* | **-** | **-** | **-** | **-** | **-** |
| 55889 | *Enterobacter aerogenes* | **-** | **-** | **-** | **-** | **-** |
| 54133 | *Enterobacter aerogenes* | **-** | **-** | **-** | **-** | **-** |
| 54063 | *Enterobacter aerogenes* | **-** | **-** | **-** | **-** | **-** |
| 54191 | *Enterobacter aerogenes* | **-** | **-** | **-** | **-** | **-** |
| 54696 | *Enterobacter aerogenes* | **-** | **-** | **-** | **-** | **-** |
| eae001 | *Enterobacter aerogenes* | **-** | **-** | **-** | **-** | **-** |
| eae002 | *Enterobacter aerogenes* | **-** | **-** | **-** | **-** | **-** |
| eae003 | *Enterobacter aerogenes* | **-** | **-** | **-** | **-** | **-** |
| eae004 | *Enterobacter aerogenes* | **-** | **-** | **-** | **-** | **-** |
| eae005 | *Enterobacter aerogenes* | **-** | **-** | **-** | **-** | **-** |
| eae006 | *Enterobacter aerogenes* | **-** | **-** | **-** | **-** | **-** |
| eae007 | *Enterobacter aerogenes* | **-** | **-** | **-** | **-** | **-** |
| eae008 | *Enterobacter aerogenes* | **-** | **-** | **-** | **-** | **-** |
| eae009 | *Enterobacter aerogenes* | **-** | **-** | **-** | **-** | **-** |
| eae010 | *Enterobacter aerogenes* | **-** | **-** | **-** | **-** | **-** |
| eae011 | *Enterobacter aerogenes* | **-** | **-** | **-** | **-** | **-** |
| eae012 | *Enterobacter aerogenes* | **-** | **-** | **-** | **-** | **-** |
| eae013 | *Enterobacter aerogenes* | **-** | **-** | **-** | **-** | **-** |
| eae014 | *Enterobacter aerogenes* | **-** | **-** | **-** | **-** | **-** |
| JM109 | *Escherichia coli* | **-** | **-** | **-** | **-** | **-** |
| DH5a | *Escherichia coli* | **-** | **-** | **-** | **-** | **-** |
| BL21 | *Escherichia coli* | **-** | **-** | **-** | **-** | **-** |
| EDL933 | *Escherichia coli* | **-** | **-** | **-** | **-** | **-** |
| 44274 | *Escherichia coli* | **-** | **-** | **-** | **-** | **-** |
| CB9767 | *Escherichia coli* | **-** | **-** | **-** | **-** | **-** |
| C1003-63 | *Escherichia coli* | **-** | **-** | **-** | **-** | **-** |
| CB176 | *Escherichia coli* | **-** | **-** | **-** | **-** | **-** |
| 315-60 | *Escherichia coli* | **-** | **-** | **-** | **-** | **-** |
| CB9720 | *Escherichia coli* | **-** | **-** | **-** | **-** | **-** |
| CB9723 | *Escherichia coli* | **-** | **-** | **-** | **-** | **-** |
| C339-54 | *Escherichia coli* | **-** | **-** | **-** | **-** | **-** |
| 637-62 | *Escherichia coli* | **-** | **-** | **-** | **-** | **-** |
| 4155 | *Escherichia coli* | **-** | **-** | **-** | **-** | **-** |
| CB9421 | *Escherichia coli* | **-** | **-** | **-** | **-** | **-** |
| CB9752 | *Escherichia coli* | **-** | **-** | **-** | **-** | **-** |
| 26W | *Escherichia coli* | **-** | **-** | **-** | **-** | **-** |
| 556-59 | *Escherichia coli* | **-** | **-** | **-** | **-** | **-** |
| 240-58 | *Escherichia coli* | **-** | **-** | **-** | **-** | **-** |
| 339-54 | *Escherichia coli* | **-** | **-** | **-** | **-** | **-** |
| CB9468 | *Escherichia coli* | **-** | **-** | **-** | **-** | **-** |
| C3813 | *Escherichia coli* | **-** | **-** | **-** | **-** | **-** |
| C288-63 | *Escherichia coli* | **-** | **-** | **-** | **-** | **-** |
| C275-53 | *Escherichia coli* | **-** | **-** | **-** | **-** | **-** |
| 539-83 | *Escherichia coli* | **-** | **-** | **-** | **-** | **-** |
| F7902-41 | *Escherichia coli* | **-** | **-** | **-** | **-** | **-** |
| M1466 | *Escherichia coli* | **-** | **-** | **-** | **-** | **-** |
| M1465 | *Escherichia coli* | **-** | **-** | **-** | **-** | **-** |
| M1459 | *Escherichia coli* | **-** | **-** | **-** | **-** | **-** |
| M1484 | *Escherichia coli* | **-** | **-** | **-** | **-** | **-** |
| M1473 | *Escherichia coli* | **-** | **-** | **-** | **-** | **-** |
| M1474 | *Escherichia coli* | **-** | **-** | **-** | **-** | **-** |
| M1475 | *Escherichia coli* | **-** | **-** | **-** | **-** | **-** |
| M1261 | *Escherichia coli* | **-** | **-** | **-** | **-** | **-** |
| M1478 | *Escherichia coli* | **-** | **-** | **-** | **-** | **-** |
| M1485 | *Escherichia coli* | **-** | **-** | **-** | **-** | **-** |
| ^a^ + indicated positive result tested by PCR; - indicated negative result tested by PCR | | | | | | |
